# Dual modality feature fused neural network integrating binding site information for drug target affinity prediction

**DOI:** 10.1038/s41746-025-01464-x

**Published:** 2025-01-28

**Authors:** Haohuai He, Guanxing Chen, Zhenchao Tang, Calvin Yu-Chian Chen

**Affiliations:** 1 State Key Laboratory of Chemical Oncogenomics, Key Laboratory of Chemical Genomics, School of Chemical Biology and Biotechnology, Peking University Shenzhen Graduate School, Shenzhen, 518055 China; 2 Artificial Intelligence Medical Research Center, School of Intelligent Systems Engineering, Sun Yat-sen University, Shenzhen, 510275 China; 3 Department of Medical Research, China Medical University Hospital, Taichung, 40447 Taiwan; 4 Department of Bioinformatics and Medical Engineering, Asia University, Taichung, 41354 Taiwan

**Keywords:** Virtual screening, Bioinformatics

## Abstract

Accurately predicting binding affinities between drugs and targets is crucial for drug discovery but remains challenging due to the complexity of modeling interactions between small drug and large targets. This study proposes DMFF-DTA, a dual-modality neural network model integrates sequence and graph structure information from drugs and proteins for drug-target affinity prediction. The model introduces a binding site-focused graph construction approach to extract binding information, enabling more balanced and efficient modeling of drug-target interactions. Comprehensive experiments demonstrate DMFF-DTA outperforms state-of-the-art methods with significant improvements. The model exhibits excellent generalization capabilities on completely unseen drugs and targets, achieving an improvement of over 8% compared to existing methods. Model interpretability analysis validates the biological relevance of the model. A case study in pancreatic cancer drug repurposing demonstrates its practical utility. This work provides an interpretable, robust approach to integrate multi-view drug and protein features for advancing computational drug discovery.

## Introduction

Accurately predicting the affinity between drugs and their biological targets is a critical step in drug discovery^1^. The affinity between a drug and its target determines the pharmacodynamic and pharmacokinetic properties of the drug^2^. However, traditional experimental measurements of drug-target binding affinities remain labor-intensive, low-throughput, and inapplicable to novel drug candidates^3^. Meanwhile, developing new medicines is a complex and resource-intensive process. It involves lengthy processes from target identification, lead compound screening, and preclinical and clinical trials. Previous studies indicate that bringing a new drug from the initial idea to market can take 10–15 years^4,5^ and cost between 400–800 million USD^6^. Therefore, the development of computational methods to accurately predict drug-target binding affinities may significantly accelerate drug discovery and reduce cost by enabling high-throughput virtual screening of large compound libraries against target proteins^7^. Among various computational approaches, molecular docking has been widely used to predict binding modes and interaction strengths between drugs and targets. Methods like AutoDock Vina^8^ and Smina^9^ employ scoring functions and search algorithms to evaluate potential binding poses. However, the computational complexity of docking methods often limits their application in large-scale screening scenarios, motivating the development of more efficient approaches.

Currently, thanks to the massive integration of biological data and the relentless efforts of researchers, artificial intelligence approaches have already demonstrated promising applications across various biomedical fields, including drug development^10^. By studying the uniqueness of different domains and tasks, researchers have constructed machine learning models tailored for various scenarios, attempting to solve problems ranging from single-cell analysis^11^, therapeutic target identification^12^, and drug synergy prediction^13,14^ to drug-drug interaction prediction^15^. Furthermore, using computational modeling, DeepMind’s AlphaFold 2 (AF2) model^16^ achieved end-to-end accurate prediction of protein 3D structures. These achievements all exhibit the tremendous contributions of AI approaches in biology and drug discovery.

For the drug-target affinity (DTA) prediction problem, many researchers have also proposed various AI approaches to model the interaction patterns between drugs and targets, thereby achieving high-precision and rapid prediction of drug-target binding affinities^17–19^. Specifically, current methods can be mainly categorized into three classes. First are pure sequence-based DTA prediction methods, which directly extract features and information from the Simplified molecular input line entry system (SMILES)^20^ strings of drugs and amino acid sequences of protein targets. They employed NLP techniques, including BiLSTM^21^, Transformer^22^, etc., for feature extraction. Ozturk et al. proposed DeepDTA^17^, the first deep learning model for drug-target affinity prediction, which utilized two independent convolutional neural networks to extract information from drug SMILES and protein amino acid sequences. MT-DTI^19^ then introduced attention mechanisms to improve model interpretability. EnsembleDLM^23^ leveraged ensemble learning methods to aggregate various sequence information, ultimately enhancing model prediction capability. Yuan et al. proposed FusionDTA^24^, which achieved high-precision attention prediction using BiLSTM and distillation models, representing the current state-of-the-art (SOTA) among pure sequence-based DTA prediction methods. However, pure sequence-based methods completely ignore the structural information of drug and target representations, such as bond information of drug molecule atoms, and residue folding and contact information of proteins.

Another class called graph-based DTA prediction methods considers the structural information of proteins and drugs to varying extents. Since drug molecules are much smaller than protein macromolecules, graph-based methods first attempt to construct molecular graphs for drugs, with atoms as nodes and bonds as edges. Meanwhile, the information on protein targets is still extracted solely from amino acid sequences in these methods. Nguyen proposed GraphDTA^18^, the first deep learning model incorporating drug molecular structure information by constructing molecular graphs and applying graph neural networks for drug structure feature extraction. Zhai et al.^25^ proposed a dynamic graph attention network to sufficiently extract features from drug molecule graphs. Yang et al. proposed MgraphDTA^26^, which utilized multi-layer graph neural and convolutional neural networks for more sophisticated extraction of drug structural and protein sequence information, achieving high-accuracy DTA prediction. This type of method that utilizes only sequence information for proteins can be categorized as the semi-graph modality-based DTA prediction method.

Recently, fully graph modality-based DTA prediction methods have been proposed to construct protein graphs, enabling structural information modeling for both drugs and proteins. These methods employ computational approaches like Pconsc4^27^ and ESM^28^ to build the contact map for proteins, and then obtain residue connectivity information by thresholding, thus constructing protein graphs with residues as nodes and contacts as edges. Zheng et al. proposed DrugVQA^29^, constructing protein graphs for drug-target interaction prediction. Chen et al. proposed GINCM-DTA^30^, enabling thorough graph modality utilization by extracting protein and drug structural information. Wang et al. proposed MSGNN-DTA^31^, which utilized ESM models^28^ for end-to-end contact map construction and further enhanced protein and drug molecular graph information utilization via Motif graphs, achieving SOTA DTA prediction performance. Among the fully graph modality-based DTA prediction methods, various approaches leverage binding site information between drug-target pairs to obtain more precise target graph representations. For instance, Torng et al.^32^ introduced a Graph-CNN framework employing a graph-autoencoder to learn fixed-size representations of protein pockets from representative druggable protein binding sites. Zhu et al. proposed DataDTA^33^, which predicts pockets from protein 3D structures and extracts their descriptors as partial input features for DTA prediction. Besides, Yousefi et al. developed BindingSite-AugmentedDTA^34^ based on their previous AttentionSiteDTI^35^ framework. This method enhances interpretability and performance by identifying key protein binding sites that contribute most to drug-target interactions. More recently, Wu et al. introduced AttentionMGT-DTA^36^, a multi-modal attention-based model for DTA prediction. This approach represents drugs and targets using molecular graphs and binding pocket graphs, respectively, further improving DTA prediction accuracy. Additionally, Zhang et al. proposed PocketDTA^37^, which leverages pre-trained models and 3D structural information of binding pockets to enhance DTA prediction performance and interpretability. Overall, previous methods attempted to incorporate more structural information for improved DTA prediction accuracy.

However, previous methods still have some limitations. Specifically, pure sequence-based DTA prediction methods completely ignore the structural information of drugs and targets. Although graph-based DTA methods incorporate structural information of proteins or drug molecules, they neglect the interaction issue. This is because drugs and proteins have different hierarchies and cannot be directly modeled together in one graph. In addition, the number of residues in a protein far exceeds the number of atoms in a drug molecule, resulting in a size discrepancy between protein and drug graphs, while drug information is also important for DTA prediction. Therefore, we need to attempt to bridge the size gap between the two graphs and facilitate information interaction. Moreover, the previous three types of methods neglected the fusion of sequence and structure modalities. Regardless of drugs or targets, they only utilized either sequence or structure information for modeling, while graph Transformer methods^38,39^ widely applied for fusing two types of information are not suitable for the DTA prediction task (see Supplementary Note 1). Besides, graph modality methods relying on predicted protein contact maps for structural information are limited by the accuracy of contact prediction methods and may not capture precise protein structural information.

**Fig. 1:**
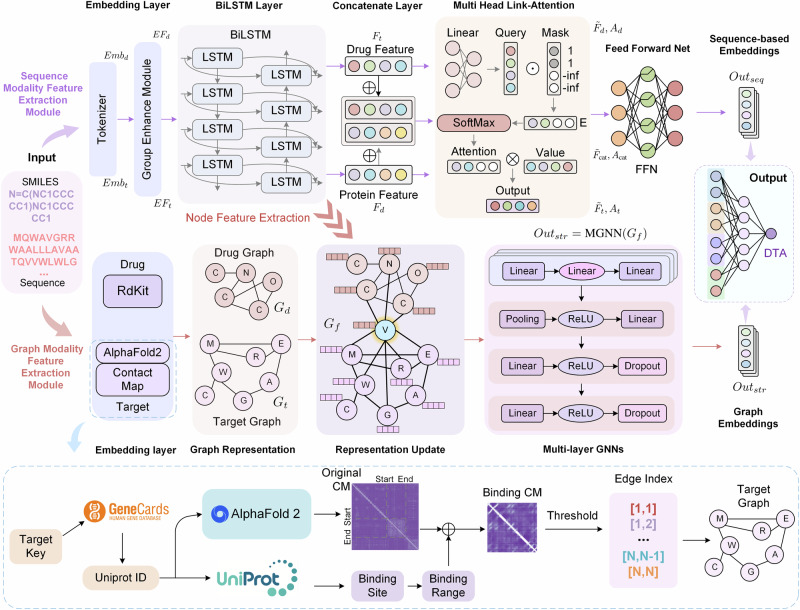
The architecture of DMFF-DTA model. The inputs to the model are the SMILES string of a drug and the amino acid sequence of its target protein. The output is a predicted binding affinity value. The model first extracts sequence-based features via the sequence modality feature extraction module, which comprises Embedding layers, bidirectional LSTM layers, and Multi-Head Link Attention components. In parallel, an innovative binding site-focused graph construction approach is used to identify and extract the binding site region from the full-protein contact map obtained through AF2. This allows the creation of a target graph focused specifically on the residues and interactions within the binding site. Additionally, a drug graph is obtained with RDKit. These are fused into a fusion graph using virtual nodes. A graph modality feature extraction module with multiple graph neural network layers then extracts graph-based features from this representation. Finally, an FFN integrates the sequence and graph modality features to output the final drug-target binding affinity prediction.

Therefore, to address those problems, we proposed a dual-modal model with feature fusion and balancing for drug-target affinity prediction (Fig. 1). It extracts features from drug SMILES and protein amino acid sequences and enables drug and target graph information interaction using graph neural networks. To resolve the lack of drug-target graph interaction and graph size imbalance in previous methods, the model focuses on the binding site contact map generated by AF2, thus alleviating the graph size discrepancy, improving interaction efficiency, and enabling more accurate modeling of the binding site. Moreover, to address the lack of simultaneously utilizing sequence and structure information in previous methods, the model constructs a BiLSTM-based sequence feature extractor and a multi-layer graph neural network module, whose interaction achieves more thorough information utilization.

In summary, our main contributions are as follows:

- We propose a dual-modal drug-target affinity prediction model with feature fusion and balancing, which contains sequence and graph modality information extraction and enables the effective fusion of sequence and structure information through innovative feature fusion and balancing, improving model accuracy in DTA prediction.
- To resolve the ineffective graph fusion due to drug-protein size discrepancy, we innovatively propose a data retrieval approach based on multiple databases and AF2 to construct binding site-focused contact maps. Target graphs constructed from these contact maps mitigate the graph imbalance issue and can model protein structural information more precisely.
- Extensive experiments, interpretability analyses, and case studies demonstrate the efficacy and potential utility value of the model. Specifically, the results showcase the model’s ability to accurately predict drug-target bindings for both previously seen and unseen drug-target pairs and indicate the model’s potential value in drug discovery fields such as drug repositioning.

## Results

### The framework of DMFF-DTA

The goal of this study is to enable thorough utilization of sequence and structure dual modality information from targets and drugs, as well as to mitigate the graph size imbalance issue between drugs and targets to achieve effective graph information fusion. Therefore, we proposed DMFF-DTA model, which incorporates a cutting-edge binding site-focused protein residue graph construction approach based on data retrieval. This reduces computational costs and facilitates the fusion of protein and drug molecular graphs. The model architecture, depicted in Fig. 1, integrates a sequence modality feature extraction module (*MFE*_*seq*_) that leverages multi-head attention and feed-forward mechanisms, along with a graph modality feature extraction module (*MFE*_*str*_) for graph-level feature extraction. Subsequently, the fused graph representation is processed through a fusion feed-forward layer to predict the binding affinity between the drug and the target.

Specifically, as illustrated in the upper half of Fig. 1, the *MFE*_*seq*_ module employs an embedding layer to extract sequence features from drugs and targets. Subsequently, a BiLSTM-based feature extractor is utilized to capture the sequential characteristics of drugs and targets. The module then incorporates a concatenation layer and Link attention method to obtain DTA embedding information based on the sequence modality.

In the *MFE*_*str*_ module, the model primarily constructs the drug graph using the Rdkit method. As shown in the lower half of Fig. 1, a binding range collection process based on AF2, GeneCard, and UniProt databases is employed to obtain the contact map of the binding range, which is then used to generate the corresponding target graph.

We introduce a virtual node to connect the drug and target graphs to minimize graph imbalance, facilitate effective information exchange between drugs and targets, and enhance the model’s interpretability through subsequent attention-based analysis. Furthermore, to align features at different levels, the atomic node features of the drug graph and the amino acid node features of the target graph are derived from the output of the feature extractor in the *MFE*_*seq*_ module. Through this workflow, the model attempts to self-model high-dimensional drug-target interaction features.

To refine and adapt the features extracted from *MFE*_*seq*_ specifically for DTA prediction tasks, the DMFF-DTA model implements a warm-up strategy. This approach involves initial training of *MFE*_*seq*_ before generating node features. Additionally, we introduce a source node method to ensure that it can identify the origin of graph nodes.

After constructing the fusion graph, *MFE*_*str*_ employs a multi-layer GNN to extract graph modality-based embeddings. Finally, the model concatenates the information from sequence and graph modalities to achieve the ultimate DTA prediction.

Detailed information about the modules mentioned above and specific content regarding the warm-up and source node strategies can be found in the Methods section.

In this work, our objective is to accurately predict the DTA value. Given a target protein *t* and a drug molecule *d*, the goal is to predict the binding affinity *y*_*t*,*d*_ between *t* and *d*, which is typically measured experimentally by the equilibrium dissociation constant (*K*_*d*_) and the half maximal inhibitory concentration (IC50). The lower the *K*_*d*_ or IC50, the stronger the binding affinity between the drug and the target. The binding affinity is usually measured by the negative logarithm of the *K*_*d*_ or IC50, which is denoted as *pK*_*d*_ or *pIC*50. In this study, we use *pIC*50 as the binding affinity measurement.

The initial inputs to our model are the amino acid sequence ${S}_{t}\in {{\mathbb{R}}}^{{L}_{t}}$ where *L*_*t*_ is the length of the sequence for target *t* and the SMILES sequence ${S}_{d}\in {{\mathbb{R}}}^{{L}_{d}}$ where *L*_*d*_ is the length of the SMILES for drug *d*. In addition, we construct the residue graph *G*_*t*_ = (*V*_*t*_, *E*_*t*_) for target *t*, where *V*_*t*_ and *E*_*t*_ refer to the set of residues and contacts, respectively. And the molecular graph *G*_*d*_ = (*V*_*d*_, *E*_*d*_) for drug *d*, where *V*_*d*_ and *E*_*d*_ denote the set of atoms and bonds, respectively.

Our model takes the target sequence *S*_*t*_, constructed residue graph *G*_*t*_, drug SMILES *S*_*d*_, and constructed molecular graph *G*_*d*_ as inputs to predict the continuous value ${\hat{y}}_{d,t}$ indicating the binding affinity between *d* and *t*. The prediction ${\hat{y}}_{d,t}$ is optimized to be as close as possible to the ground-truth experimental measurement *y*_*d*,*t*_ by minimizing a regression loss function during model training.

### DMFF-DTA can accurately predict drug-target affinity

**Fig. 2:**
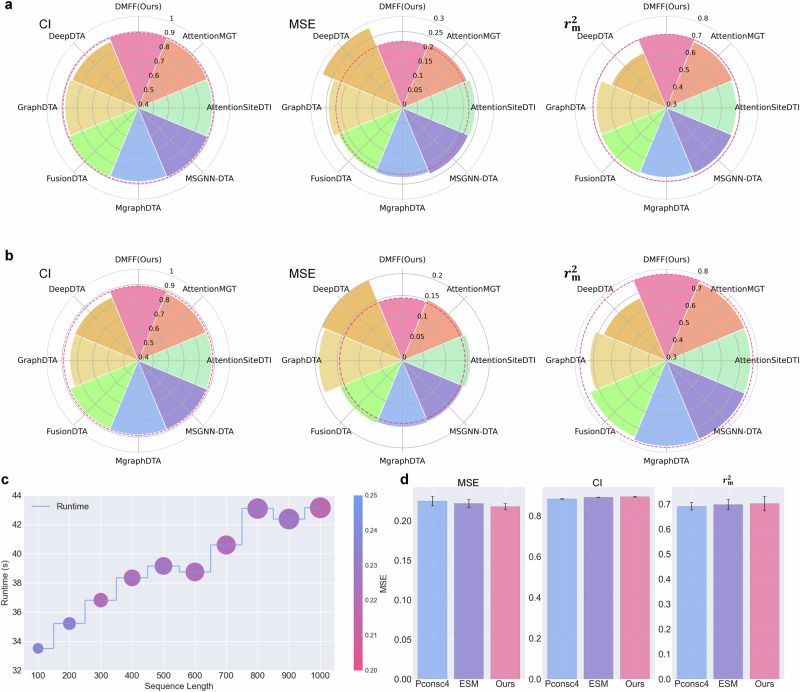
Performance comparison and analysis of DMFF-DTA. **a**, **b** Polar bar charts depicting DMFF-DTA against other SOTA models on three evaluation metrics for the Davis (**a**) and KIBA (**b**) datasets. The height of each bar signifies the metric value for the corresponding model. In each polar plot, a dashed line of the same color as the best-performing model extends at the level of that model’s metric value across the chart, thereby facilitating an intuitive visual comparison of the best model against the others. **c** A step line chart overlaid with a sized scatter plot showing the relationship between protein sequence length and model training runtime. The *x*-axis denotes the binding site length interval, while the *y*-axis indicates the runtime per training epoch. For each interval length, the color of the scatter points represents the final MSE on the test set, as depicted in the accompanying color bar. Additionally, the size of the scatter points indicates the GPU memory consumption during training for that interval length. **d** Bar charts illustrating the performance of three graph construction techniques across three evaluation metrics, emphasizing the superior precision of our AF2-based contact map generation.

To evaluate the accuracy of drug-target affinity prediction by DMFF-DTA, we compared it against several classic DTA prediction methods: DeepDTA, GraphDTA, and two DTA prediction methods based on binding-site information, AttentionSiteDTI and AttentionMGT, and SOTA methods, FusionDTA, MgraphDTA, and MSGNN-DTA, from three categories - Pure sequence-based, Semi-graph modality-based, and Fully graph modality-based DTA prediction methods. Therefore, we selected seven previous methods in total with available official code implementations. For an intuitive visualization of DMFF-DTA’s comparative performance, Fig. 2 presents a comprehensive view.

The detailed experimental settings are described in Methods section. To enable fair comparison, all methods were evaluated using the same experimental settings.

Figure 2a, b depict radar charts contrasting DMFF-DTA’s performance with other methods across various metrics on different datasets, with the best model in each metric encircled. The radar charts visually underscore DMFF-DTA’s superior DTA prediction accuracy, achieved through effective utilization of drug-target sequence and structure information. As presented in Fig. 2a and Supplementary Table 1, our model outperforms other SOTA models on the Davis dataset across all three metrics. Specifically, DMFF-DTA reduces the MSE by 3.6% (from 0.226 to 0.218) and increases the CI by 0.3% (from 0.891–0.894) compared to the second-best method, FusionDTA. We conducted the *t*-test and the result shows the statistical significance of these improvements in MSE and CI(*P* < 0.05). Furthermore, DMFF-DTA attains the highest ${r}_{m}^{2}$ value of 0.702, surpassing all other approaches, including the binding site-based method AttentionMGT. This underscores the efficacy of our model’s sequence and graph information fusion.

As presented in Fig. 2b and Supplementary Table 2, the superior performance is consistent on the KIBA dataset, with DMFF-DTA outperforming on all metrics. It decreases the MSE by 3.4% (from 0.149 – 0.144) and improves the CI by 0.5% (from 0.885–0.889) relative to the second-best MSGNN method. These enhancements are statistically significant (*P* < 0.05). Notably, our model achieves a new SOTA ${r}_{m}^{2}$ metric of 0.773, outperforming the second-best method, MgraphDTA. Collectively, these results underscore DMFF-DTA’s capacity to leverage graph structure information for more accurate DTA prediction.

### DMFF-DTA balances performance and cost

To evaluate the efficacy of our proposed protein binding site graph construction approach using AF2 and data retrieval, we conducted comparative experiments focusing on both performance and computational costs.

First, we examined the training time per epoch on the Davis dataset, GPU memory consumption, and final DTA prediction performance with different binding range settings in our model. The results in Fig. 2c and Supplementary Table 3 show that as the set binding range increases, the required training time also rises, with noticeable jumps from 300–400 and 700–800. Concurrently, the memory overhead grows consistently with larger binding ranges. These trends are expected since longer ranges lead to more nodes and edges in the target graphs, increasing computational expenses for the MGNNs. Besides, The sequence lengths also affect the cost of LinkAttention. However, examining the model performance reveals that the MSE stays fairly steady for binding ranges over 300. This phenomenon implies that reducing the range enables the model to disregard superfluous information and concentrate on creating associations between drugs and target binding sites. At the same time, it facilitates a more balanced integration of information from the drug and target graphs.

To further demonstrate the balance between performance and cost achieved by DMFF-DTA, we compared it with other state-of-the-art models. Supplementary Table 4 shows that DMFF-DTA achieves the lowest MSE (0.218) while maintaining a reasonable balance in runtime and GPU memory consumption. Specifically, DMFF-DTA’s runtime (36.80 s) and GPU consumption (17,681 MB) are significantly better than fully graph-based methods like MSGNN-DTA (32.78 s, 22,340 MB) and binding site-based AttentionMGT-DTA (321.59 s, 21,726 MB). MgraphDTA and FusionDTA, which are based on semi-graph modality and pure sequence-based DTA prediction methods, respectively, show lower GPU consumption compared to fully graph modality-based DTA prediction methods like DMFF. This lower GPU consumption is mainly because these methods do not use target graphs in their approaches. However, their performance in terms of MSE (0.228 for MgraphDTA and 0.226 for FusionDTA) is inferior to that of DMFF-DTA (0.218). Additionally, MgraphDTA’s lower runtime can be attributed to its lack of attention mechanisms, which might result in reduced interpretability compared to methods that use attention mechanisms. Overall, these results highlight the effective balance between performance and computational cost achieved by DMFF-DTA. It outperforms other methods in prediction accuracy while maintaining reasonable computational requirements. This combination of high accuracy and efficiency makes DMFF-DTA a valuable tool for researchers in drug-target affinity prediction.

Additionally, to verify the accuracy of our approach, we compared our AF2-based contact map generation methods against Pconsc4 and ESM, which were employed by previous SOTA DTA methods for contact map construction. Specifically, we swapped different contact map generation techniques and evaluated model performance on the Davis dataset. Figure 2d demonstrates our AF2-based contact maps enable superior performance across all three metrics. This validates the higher precision of AF2 for contact map generation compared to prior techniques, allowing the model to acquire more accurate protein structure information and thus better model target graphs.

To further validate the effectiveness of the AF2 method, we conducted additional experiments using MSGNN-DTA, the SOTA Fully graph modality-based DTA Prediction Method. We modified its contact map construction technique to compare the effects of different construction methods. As shown in Supplementary Table 5 and Supplementary Fig. 1, MSGNN-DTA exhibits a trend consistent with DMFF, where our AF2-based contact maps achieve superior performance across all three metrics. The result further confirms the effectiveness of the AF2-based approach.

In summary, the experiment results proved our proposed innovations for protein binding site modeling, using AF2 and data retrieval, reduce training costs while maintaining performance, and also enable more precise extraction of target structural information through accurate AF2-based contact maps.

### Evaluating model generalization performance on unseen drugs and ttargets

In previous experiments, model performance was compared using five-fold cross-validation. However, random data splitting for DTA prediction risks falsely elevated performance, as targets or drugs in the test set may be seen during training, causing information leakage^40^. In real drug discovery, a model’s ability to generalize to unseen drugs and targets is more valued. Hence, we designed experiments to evaluate our model’s generalization on novel drugs and targets.

Specifically, three scenarios were defined: unseen drugs, unseen targets, and completely unseen. For unseen drugs, the drug set was split into training, validation, and testing subsets, ensuring no drugs present in training drug-target interactions appear in validation or testing. This tests generalization to new drugs. The unseen target scenario is similar, partitioning targets instead of drugs. The all unseen scenario further ensures no overlapping drugs or targets amongst train, validation, and test sets, maximally challenging generalization.

We conducted these experiments on the Davis and KIBA benchmarks, comparing our model against the 5 previously described SOTA methods. For fairness, the same data-splitting strategy was applied to each technique, with five repeats reporting average metric values and standard deviations.

The results in Tables 1 and 2 show our model achieves superior performance over contrastive methods on both datasets across all three scenarios. On the unseen drug scenario, our model improved average MSE, CI, and ${r}_{m}^{2}$ by 7.2% and 4.4% over the second-best method. For the unseen target scenario, our model achieves average gains of 8.5% and 9.0% over the next best approach. In the most challenging all unseen scenario, our method demonstrates significant boosts of 10.0% and 9.2% in average MSE, CI, and ${r}_{m}^{2}$ compared to other models. This demonstrates significant enhancements versus other models under more realistic generalization testing.

**Table 1:** Performance comparison (average ± std) between our model and SOTA methods on unseen drugs, unseen targets, and completely unseen scenarios across the Davis dataset

Scenario	Method	MSE *↓*	CI*↑*	${r}_{m}^{2}\uparrow$
Unseen drug	GraphDTA	0.920(0.029)	0.678(0.036)	0.160(0.019)
	FusionDTA	0.581(0.094)	0.737(0.012)	0.187(0.034)
	MgraphDTA	0.563(0.065)	0.729(0.022)	0.192(0.021)
	MSGNN-DTA	0.560(0.075)	0.731(0.036)	0.186(0.058)
	DMFF(Ours)	**0.548(0.063)**	**0.742(0.037)**	**0.228(0.073)**
Unseen target	GraphDTA	0.510(0.086)	0.729(0.012)	0.154(0.014)
	FusionDTA	0.364(0.021)	0.826(0.011)	0.435(0.023)
	MgraphDTA	0.359(0.023)	0.813(0.008)	0.425(0.028)
	MSGNN-DTA	0.361(0.053)	0.816(0.018)	0.430(0.039)
	DMFF(Ours)	**0.330(0.047)**	**0.840(0.014)**	**0.501(0.044)**
All unseen	GraphDTA	0.968(0.096)	0.579(0.017)	0.026(0.016)
	FusionDTA	0.876(0.091)	0.645(0.043)	0.072(0.048)
	MgraphDTA	0.874(0.090)	0.636(0.021)	0.071(0.041)
	MSGNN-DTA	0.791(0.078)	0.650(0.053)	0.076(0.064)
	DMFF(Ours)	**0.759(0.071)**	**0.655(0.050)**	**0.095(0.076)**

*↑*/*↓* indicates that the larger/smaller the metrics, the better the model performance. Bold indicates the best performance, and underline indicates the second best for each metric. DMFF-DTA achieves superior performance over other methods in all scenarios, demonstrating its strong generalization capabilities.

**Table 2:** Performance comparison (average ± std) between our model and SOTA methods on unseen drugs, unseen targets, and completely unseen scenarios across the KIBA dataset

Scenario	Method	MSE *↓*	CI*↑*	${r}_{m}^{2}\uparrow$
Unseen drug	GraphDTA	0.471(0.047)	0.713(0.002)	0.342(0.007)
	FusionDTA	0.429(0.031)	0.748(0.005)	0.364(0.012)
	MgraphDTA	0.425(0.047)	0.746(0.002)	0.366(0.016)
	MSGNN-DTA	0.426(0.043)	0.747(0.003)	0.358(0.022)
	DMFF(Ours)	**0.408(0.036)**	**0.753(0.006)**	**0.397(0.020)**
Unseen target	GraphDTA	0.469(0.089)	0.610(0.035)	0.368(0.057)
	FusionDTA	0.439(0.062)	0.685(0.032)	0.390(0.067)
	MgraphDTA	0.435(0.055)	0.674(0.028)	0.382(0.047)
	MSGNN-DTA	0.438(0.061)	0.683(0.025)	0.399(0.054)
	DMFF(Ours)	**0.410(0.063)**	**0.748(0.053)**	**0.446(0.064)**
All unseen	GraphDTA	0.676(0.113)	0.601(0.030)	0.149(0.067)
	FusionDTA	0.587(0.086)	0.641(0.023)	0.193(0.053)
	MgraphDTA	0.590(0.094)	0.626(0.028)	0.182(0.012)
	MSGNN-DTA	0.581(0.079)	0.648(0.038)	0.180(0.021)
	DMFF(Ours)	**0.567(0.082)**	**0.667(0.035)**	**0.236(0.037)**

*↑*/*↓* indicates that the larger/smaller the metrics, the better the model performance. Bold indicates the best performance, and underline indicates the second best for each metric. DMFF-DTA achieves superior performance over other methods in all scenarios, demonstrating its strong generalization capabilities.

To rigorously assess the statistical significance of our model’s performance improvements, we conducted *t*-tests comparing our results with the second-best method for each scenario and dataset. For the Davis dataset, our model demonstrated statistically significant improvements in CI and ${r}_{m}^{2}$ (*p* < 0.05) compared to the second-best method in the unseen drug scenario. In contrast, the improvement in MSE was not statistically significant. In the unseen target scenario, our model achieved statistically significant improvements across all metrics (MSE, CI, and ${r}_{m}^{2}$) with *p* < 0.01, indicating a robust enhancement in performance. However, in the all unseen scenario for Davis, the improvements across all three metrics did not reach statistical significance, likely due to increased performance variability, reflected in larger standard deviations under this most challenging condition.

For the KIBA dataset, our model showed more consistent statistically significant improvements. In the unseen drug scenario, statistically significant enhancements were observed across all metrics (MSE, CI, and ${r}_{m}^{2}$) with *p* < 0.05. The unseen target scenario yielded even stronger results, with CI showing significance at *p* < 0.01, and MSE and ${r}_{m}^{2}$ at *p* < 0.05. Notably, even in the challenging all unseen scenario for KIBA, our model maintained statistically significant improvements in CI and ${r}_{m}^{2}$ (*p* < 0.05), although the improvement in MSE did not reach statistical significance. These statistical analyses provide strong evidence for the superior performance of our model, particularly in scenarios involving unseen drugs and targets.

Overall, these results indicated our model builds more faithful representations of drug-target interactions and target structure to enable highly accurate DTA prediction even for unseen drugs and targets. Such generalization abilities lend our model stronger potential for practical drug discovery applications.

### The components of DMFF-DTA contribute to the predictive performance

To verify the contribution of each component in the model to the accurate drug-target affinity prediction ability, we decomposed different components of the model and conducted ablation experiments. Specifically, we removed different components of the model while keeping other parts, and then evaluated the model performance using the data splitting and 5-fold cross-validation methods. The detailed settings for these experiments are described in Methods section.

Regarding the model architecture, we tested the following components: GEM, LinkAttention module, the whole *MEF*_*seq*_ module, and the entire *MEF*_*str*_ module. For each module, we removed the corresponding part from the full model while keeping the other modules unchanged. For example, W/o *MEF*_*str*_ represents that we do not use any graph modality information, but directly extract features and make predictions on the sequence modality of the drug-target pair using *MEF*_*seq*_. Regarding the training strategy and graph construction, we tested the following components: Virtual Node, Source feature, and Warm Up strategy. For each of the above modules, we removed the corresponding strategy while maintaining the complete model architecture. For example, W/o Virtual Node represents not using the Virtual node to connect the target and drug graphs, but using two independent MGNNs to extract and aggregate features on the target and target graphs separately.

As shown in Table 3, the full model achieved the best performance across all metrics, which demonstrates that each module constituting the model contributes to its DTA prediction capability.

**Table 3:** Ablation evaluation results for different components of DMFF-DTA on the Davis dataset

	MSE	CI	${r}_{m}^{2}$
W/o GEM	0.224	0.889	0.701
W/o LinkAttention	0.234	0.877	0.699
W/o Virtual node	0.230	0.886	0.695
W/o Source feature	0.222	0.891	0.699
W/o Warm up	0.231	0.885	0.696
W/o *MEF*_*seq*_	0.387	0.797	0.478
W/o *MEF*_*str*_	0.233	0.880	0.685
Full model	**0.218**	**0.894**	**0.702**

“W/o” means removing the corresponding component from the full model. The Full model is proposed DMFF-DTA. Bold indicates the best performance, and underline indicates the second best for each metric.

Removing the entire *MEF*_*seq*_ module caused a substantial performance decline, significantly reducing the model’s DTA prediction accuracy. The result suggests that other modules likely depend on the *MEF*_*seq*_ module to extract meaningful node features. Apart from the *MEF*_*seq*_ module, removing the entire *MEF*_*str*_ module or the LinkAttention module had the greatest impact on model performance, with an overall performance drop of >3%, respectively. Besides, removing the virtual nodes and the Warm Up strategy led to overall performance drops of 2.4% and 2.6%, respectively. Moreover, the absence of the other two components, GEM and Source feature, resulted in overall performance drops of ~1%, respectively.

The results highlight that the *MEF*_*seq*_ module’s extraction of meaningful node features is critical for the model’s performance. Furthermore, they indicate that both extracting structural modality information and capturing global sequence information are crucial. In addition, both of the virtual nodes enabled the fusion and interaction of the two graph modalities, and the Warm Up strategy ensured effective node features, making important contributions to model performance. Furthermore, GEM’s integration and enhancement of sequence modality features and the Source feature’s provision of prior information to the fusion graph also had positive effects on model performance.

In summary, the ablation study results validate the rationality and effectiveness of DMFF-DTA’s component design. The performance drops when removing different components prove that each module contributes positively to the model’s DTA prediction ability.

### DMFF-DTA is highly interpretable

Understanding the decision-making process of computational models is crucial, especially in drug discovery, where interpretability can validate the biological relevance of predictions. In this subsection, we analyze the attention mechanisms of our model, which provide insights into its predictive behavior by highlighting the importance of specific regions within the protein sequences for drug-target affinity prediction.

**Fig. 3:**
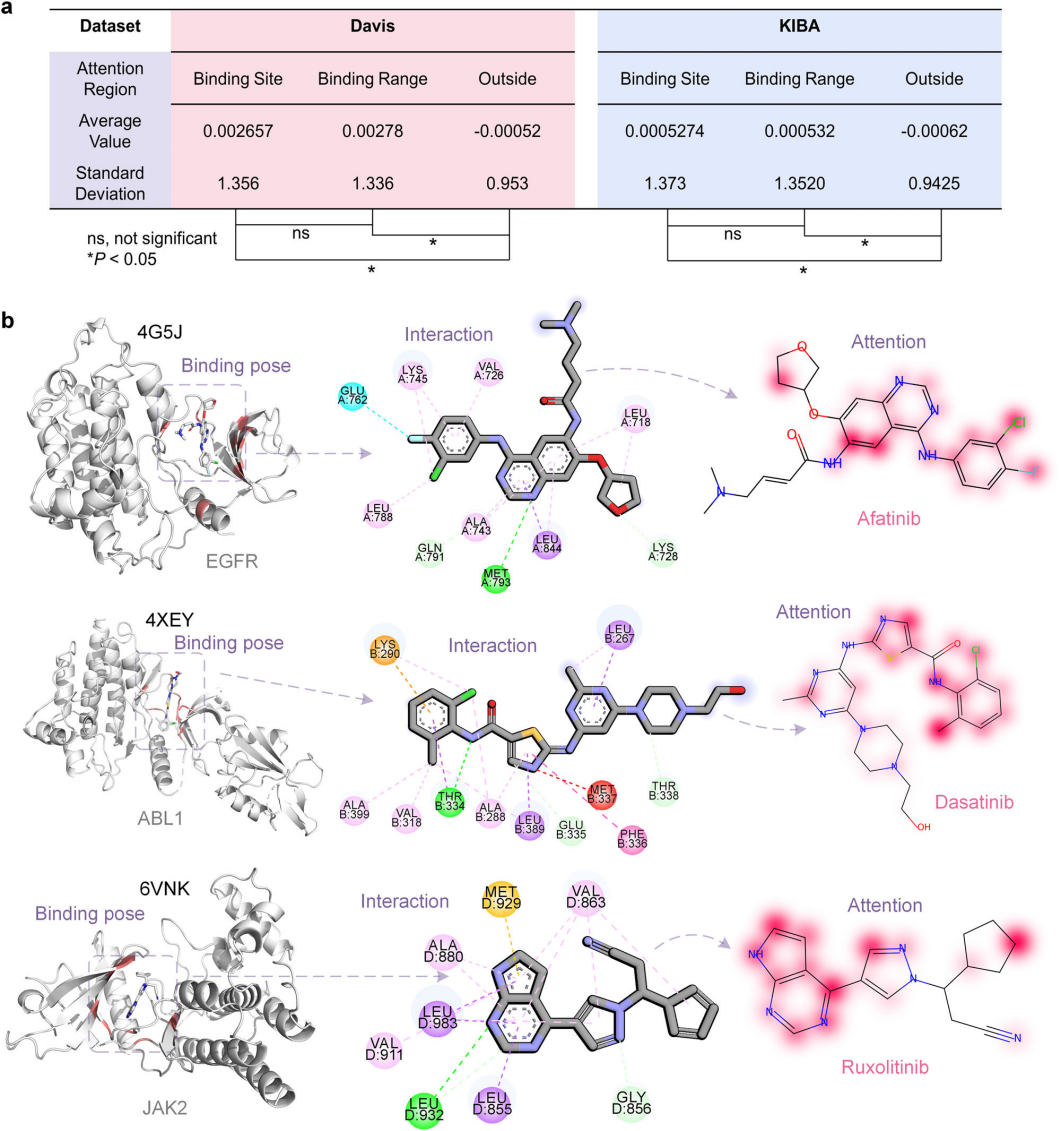
Interpretable analysis of DMFF-DTA model. **a** A statistical table showing the average and standard deviation of attention weights output by the model across the Binding Site, Binding Range, and Outside of Binding Range regions for Davis and KIBA datasets. Below is the *t*-test result comparing attention distributions between different regions. “ns” indicates no significant difference, while asterisks denote significant differences (*p* < 0.05) between regions. **b** Visual cases of drug-target complexes from PDB entries 4G5J, 4XEY, and 6VNK. Each row shows one case, with a 3D visualization of the complex on the left indicating the binding pose. Red residues are those assigned high attention weights by the model. The middle column illustrates interactions between the target and drug molecule. Attention weights output by the model for the drug molecule are shown on the right, with deeper colors representing higher attention values.

We present a comprehensive statistical analysis of attention weights for protein sequences within the Binding Site, Binding Range, and Outside regions across both Davis and KIBA datasets. As depicted in Fig. 3a, a significant elevation is evident in attention values for regions within the binding site and range compared to those outside (*t*-test, *p* < 0.05). This suggests a correct emphasis on areas directly involved in drug interactions. Furthermore, the negative weights assigned to regions outside the binding influence demonstrate the model’s proficiency in discriminating between relevant and irrelevant areas. Notably, despite distinct attention on the binding site and range, no statistically significant difference exists between their attention values across both datasets. The model’s capacity to focus on key binding regions is likely facilitated by our novel binding site contact map approach, which provides spatial binding information to guide the model to preferentially attend to interaction-critical areas during training. Quantitative analysis of attention distributions validates that the model learns to emphasize biologically relevant regions for accurate drug-binding affinity predictions.

While the previous quantitative analysis validates the overall attention distribution, visual inspection of individual cases provides a further intuitive demonstration of the model’s interpretability. Figure 3b shows three PDB complexes: 4G5J (EGFR with Afatinib)^41^, 4XEY (ABL1 with Dasatinib)^42^, and 6VNK (JAK2 with Ruxolitinib)^43^, to elucidate the model’s interpretability on an individual case basis. Each example comprises a 3D binding pose, depicting the spatial relationship between the drug and its target protein, and a 2D interaction diagram that details specific interactions, such as hydrogen bonds and hydrophobic contacts, between the drug and key amino acids. Furthermore, the attention visualization on the drug molecules accentuates the regions deemed crucial for binding by the model, with the intensity of the color corresponding to the attention weight. The drugs and proteins in all three examples engage in multiple interactions. We observed that functional groups such as O, N, Cl, and F in the drug molecules receive higher weights, as do phenyl ring structures. Most notably, the positions on the drugs where protein amino acids exert their effects also garner higher attention weights. This visualization not only corroborates the biological plausibility of the model’s predictions but also offers a clear delineation of the model’s focus, aligning with established interaction sites.

The integration of attention-based interpretability with structural analysis of drug-target complexes provides a comprehensive understanding of the model’s predictive capabilities. Quantitative analysis of attention distributions confirms that the model focuses on biologically relevant binding regions. Additionally, visualizing attention weights directly on 3D drug-target structures aligns with known binding sites and interactions. Through these analyses, we can better understand the rationales behind the model’s predictions, validating the biological relevance of its attention patterns. Therefore, this interpretability analysis reinforces confidence in the reliability of the model’s predictions, supporting the application of our interpretable deep learning approach for drug discovery tasks requiring trustworthy and rational predictions.

### Case study on pancreatic cancer verified the utility of DMFF-DTA

Pancreatic cancer represents an aggressive disease with poor prognosis and limited treatment options^44^. As the third leading cause of cancer-related deaths, new therapeutic strategies for this malignancy are urgently needed. In this case study, we utilized DMFF model for the repurposing of drugs in the context of pancreatic cancer. Through a systematic analysis combining pathway mapping and evaluation of physicochemical properties, including absorption, distribution, metabolism, excretion, and toxicity (ADMET), we identify promising candidates for repurposing.

**Fig. 4:**
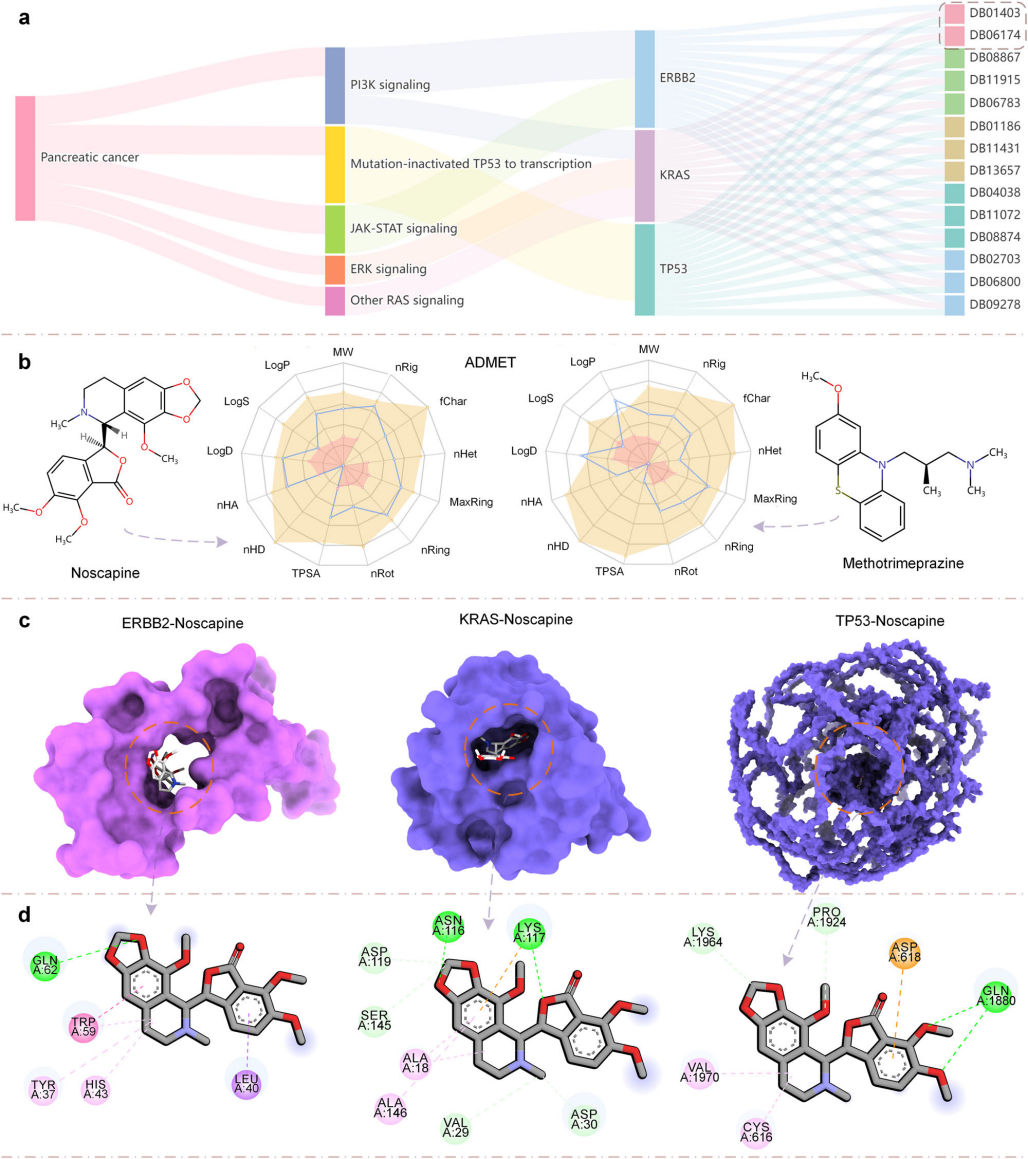
Drug repurposing analysis for pancreatic cancer. **a** Relationship mapping of pancreatic cancer, associated pathways, targets, and drugs for repurposing, derived through KEGG database analysis. **b** Chemical structures and physicochemical properties predictions for selected drugs Noscapine (DB01403) and Methotrimeprazine (DB06174), with desirable physicochemical properties indicated by the radar plot’s coverage within the shaded area. **c** 3D binding poses of Noscapine with three targets generated by the CB-Dock2 server. **d** 2D interaction diagrams for the docking poses, detailing the interactions between Noscapine and the target residues. MW (Molecular Weight): Represents the mass of a molecule, influencing drug distribution and elimination; LogP: A measure of lipophilicity, playing a role in drug absorption and distribution; LogS: An indicator of solubility, crucial for determining oral bioavailability; LogD: Reflects the distribution coefficient, shedding light on drug partitioning within the body;nHA (Number of Hydrogen Acceptors) & nHD (Number of Hydrogen Donors): Denote a molecule’s hydrogen bonding capability, affecting solubility and receptor binding; TPSA (Topological Polar Surface Area): Correlates with drug transport properties; nRot (Number of Rotatable Bonds): Influences oral bioavailability; nRing (Number of Rings), MaxRing (Maximum Size of Rings), nHet (Number of Heteroatoms), fChar (Formal Charge), and nRig (Number of Rigid Bonds): These structural properties play roles in drug-receptor interactions and overall pharmacokinetics.

Figure 4a illustrates the associations between pancreatic cancer, pathway information, pathogenic targets, and drugs identified for repurposing. First, we retrieved the pathway collection for Pancreatic Cancer (ID: map05212) from the KEGG database^45^. Next, we analyzed each network to identify explicit pathogenic targets contained within them. KRAS was present in “ERK signaling”, “PI3K signaling”, “Other RAS signaling” networks; ERBB2 target existed in “PI3K signaling”, “JAK-STAT signaling”; CDKN2A existed in the “Cell cycle” network; and TP53 was present in the “Mutation-inactivated TP53 to transcription” network. We then utilized the ChEMBL database^46^ to gather drug affinity data for small molecule compounds against the pathogenic targets present in the networks. Since no related data existed for CDKN2A (CHEMBL4680027), it was excluded from further analysis. Ultimately, pathway information, target-drug affinity data, and linkage to pancreatic cancer were collected for three pathogenic targets: ERBB2, KRAS, and TP53. We gathered 2889 drug-target affinity (DTA) samples from ChEMBL for these targets. Subsequently, we fine-tuned the pre-trained DMFF model using these pancreatic cancer target data. The fine-tuned model was then used to predict binding affinities against the targets mentioned above for 2509 FDA-approved drugs. It is worth noting that we verified that these FDA-approved drugs had no overlap with the compounds used in our training and fine-tuning datasets, ensuring a fair assessment of our model’s generalization ability.

To demonstrate the effectiveness of DMFF, we compared its performance with other SOTA DTA models on this pancreatic cancer dataset. Supplementary Table 6 shows that DMFF achieves superior prediction accuracy compared to other models. Specifically, DMFF obtains the lowest MSE of 0.212, the highest CI of 0.881, and the highest ${r}_{m}^{2}$ of 0.842. These results indicate that DMFF can capture the complex interactions between drugs and targets more accurately than existing methods, which is crucial for reliable drug repurposing predictions.

This resulted in an FDA drug repurposing list with DrugBank IDs of candidates shown in the rightmost column of Fig. 4a. Among the drugs evaluated for repurposing, Noscapine (DB01403) and Methotrimeprazine (DB06174) are highlighted, with Noscapine being the focus due to its optimal physicochemical profile suggested by the complete attribute region in the shaded radar plot (Fig. 4b).

The physicochemical properties of the two drugs were then calculated using the ADMETlab 2.0 web server^47^. In Fig. 4b, the chemical structures of Noscapine and Methotrimeprazine are presented alongside their physicochemical properties prediction outcomes displayed in a spider chart format. The distribution of Noscapine’s physicochemical properties falls entirely within the shaded area, signifying excellent performance, whereas Methotrimeprazine shows larger LogP and LogD values, indicating potential issues with lipid solubility and distribution.

Figure 4c depicts the 3D binding poses of Noscapine with three targets, obtained through molecular docking on the CB-Dock2 web server^48^. The resulting poses suggest the interactions within the binding sites of the targets. Subsequently, Fig. 4d provides a detailed 2D interaction diagram for each docking pose, highlighting the specific interactions such as hydrogen bonds and hydrophobic contacts between Noscapine and key amino acid residues.

Through this case study, we demonstrate the application of our DMFF model in identifying and validating potential drugs for repurposing in pancreatic cancer treatment. The multi-step analysis encompasses pathway mapping, target-drug affinity prediction, physicochemical properties profiling, and molecular docking for validation. While focused on pancreatic cancer, the approach is broadly applicable across diseases to uncover therapeutic rediscovery opportunities. The DMFF model efficiently contributes to prioritizing FDA-approved drugs by providing accurate affinity predictions, which are then integrated with other critical factors such as target relevance, pathway analysis, and drug properties. This case highlights the strengths of DMFF in accelerating the initial screening phase of drug repurposing efforts, serving as a crucial component in a robust, multi-faceted methodology from target to hit identification.

## Discussion

In this study, we proposed DMFF-DTA, a dual-modal neural network model with feature fusion and balancing for accurate drug-target affinity prediction. The model effectively integrates sequence and graph structure information from both drugs and proteins through innovative feature extraction and fusion modules. To address the graph imbalance issue between drugs and proteins, we introduced a novel binding site-focused graph construction approach based on AF2 structure predictions and data retrieval. This enables more balanced and efficient graph neural network-based modeling of drug-protein interactions.

Experiments demonstrate our model achieves superior performance over previous SOTA methods on two benchmark datasets. The model also exhibits excellent generalization capabilities on novel unseen drugs and targets. Furthermore, we performed a comprehensive interpretability analysis and a case study for drug repurposing in pancreatic cancer treatment, showcasing the model’s practical applicability. The model’s interpretability analysis further strengthens the confidence in its predictions, ensuring that the decision-making process aligns with biological relevance.

This work makes significant contributions towards advancing computational drug discovery through dual-modal neural networks for drug-target affinity prediction. Our model provides a powerful and interpretable approach to enable more accurate and rapid virtual screening, drug candidate optimization, and drug repurposing.

Introducing virtual nodes in our model serves multiple crucial purposes while maintaining the structural integrity of both drug and protein graphs. Our approach bridges the information flow between these hierarchically distinct graphs without compromising their inherent structures. This effect is achieved by adding connections through virtual nodes rather than altering existing edges, thus preserving the original graph topologies. The effectiveness of this method is corroborated by our ablation studies, which demonstrate performance improvements when virtual nodes are incorporated. Moreover, this design enhances the model’s interpretability. By analyzing the attention values associated with the virtual nodes, we can derive attention weights for each amino acid in the protein and each atom in the drug molecule. Our interpretability analysis proves that the model can learn key binding sites, providing valuable insights into the specific structural elements that contribute most significantly to drug-target interactions. This information could potentially guide future drug design efforts.

Besides, the virtual node approach offers a unique perspective on modeling drug-target interactions. This method can be seen as an abstraction of the binding pocket concept in molecular biology. Just as a binding pocket serves as a specific region where a drug molecule interacts with a target protein, our virtual node is a computational representation of this interaction space. It allows for focused information exchange between the drug and protein graph without oversimplifying their complex relationships.

Our decision to use a virtual node instead of directly connecting all protein nodes to all drug nodes is based on both computational efficiency and biological relevance. Direct all-to-all connections would result in a dense graph structure, significantly increasing computational complexity and potentially introducing noise in the interaction modeling. The virtual node approach strikes a balance, allowing for comprehensive information flow while maintaining a manageable graph structure.

It is worth noting that previous methods often simplified drug representations to a single node when connecting to protein graphs^49^, or did not implement drug-target information exchange at the graph level^26,32,36^. Our approach, while still having room for improvement, attempts to facilitate information exchange between drug and target graph structures while preserving the structural information of the drug. The virtual node is a computational construct that enables this exchange without imposing unrealistic constraints on the molecular structures.

Future work could focus on refining this approach to more closely align with specific biological mechanisms of drug-target interactions. This might involve developing dynamic adjustment methods to optimize the balance between original graph information and cross-graph interactions or exploring alternative connection schemes that better reflect the physical reality of molecular binding processes. While our current method effectively captures essential interaction patterns, we recognize the potential for even more biologically grounded approaches and see this as an important direction for future research.

Besides, it is important to note that while our DMFF model provides valuable insights through accurate drug-target affinity predictions, drug repurposing decisions cannot be based solely on these predictions. Affinity values are a crucial starting point, but a comprehensive approach to drug repurposing must consider multiple factors. These include but are not limited to the biological relevance of the target in the disease context, the drug’s pharmacokinetic and pharmacodynamic properties, potential off-target effects, and the complex interplay of molecular pathways involved in the disease. Our case study demonstrates how DMFF can be integrated into a multi-faceted approach, where affinity predictions serve as an initial filter to identify promising candidates. These candidates then undergo further evaluation through additional computational and experimental methods. This integrated strategy leverages the strengths of our model while acknowledging the complexity of drug repurposing decisions. Future work could focus on developing more comprehensive computational frameworks that integrate affinity predictions with other vital factors to provide a more holistic assessment of drug repurposing potential.

Furthermore, we acknowledge that our DMFF-DTA model, which uses UniProt-annotated binding site information, may have limitations in predicting drug-target affinity for inhibitors with new or unannotated binding sites. This could potentially restrict its application to type 1 and 2 kinase inhibitors that bind to ATP or substrate sites. To address the issue, future work should focus on developing more comprehensive and accurate methods for obtaining binding site information, enhancing the model’s ability to fit diverse interactions.

While DMFF utilizes binding range information, it is primarily used to reduce computational cost and improve efficiency. Our experiments demonstrate that the model can effectively predict drug-target affinity even without binding range information. In contrast, other binding site-based methods rely heavily on constructing binding graphs through predictive or distance-based approaches. This dependence on prior binding site information potentially limits their generalization capabilities. Our comparative study shows that DMFF outperforms binding site-based methods in both known and unknown binding pocket scenarios (Supplementary Note 2), highlighting its robustness and adaptability to various drug-target interaction contexts.

While attention mechanisms provide insights into the model’s predictions, recent studies have questioned the validity of attention as the explanation for deep learning systems^50^. Although we performed a quantitative analysis to mitigate unreliability, it is important to exercise caution when interpreting attention weights as true model explainability. Further research on more rigorous explanation methods is still needed in this field. Looking ahead, directly predicting potential binding sites could further enhance the efficiency and applicability of the DTA prediction task. Moreover, expanded experiments on diverse protein families would better validate generalization.

Overall, the DMFF-DTA model emerges as a powerful tool in the drug discovery arsenal, offering significant contributions to the acceleration and cost-efficiency of the drug development process. Its robust performance, interpretability, and generalization capabilities underscore its potential for practical applications in the biomedical field.

## Methods

### Drug and target representations

The initial inputs to the model are the amino acid sequences of targets *S*_*t*_ and the SMILES strings of drugs *S*_*d*_. Through tokenizers, the above texts are split into two token sets *Token*_*t*_ and *Token*_*d*_. The target tokenizer is residue-level, with its vocabulary containing single-letter token of each type of amino acid. The drug tokenizer ensures atom-level tokenization, which means each atom in a SMILES string has an independent token.

For the graph-level representations, the model first constructs the drug molecular graph *G*_*d*_ = (*V*_*d*_, *E*_*d*_), where *V*_*d*_ refers to the atoms in the drug graph. *E*_*d*_ is obtained through the RDKit^51^ library based on the bonding relationships between atoms in the drug molecule. Then, the model constructs the target graph representation *G*_*t*_ = (*V*_*t*_, *E*_*t*_). Specifically, the Uniprot ID corresponding to the target is obtained through the GeneCards database^52^ based on the *Key*_*target*_. The AF2 Database^53^ is then used to query and retrieve the protein structure for the Uniprot ID. The structures in the AF2 Database were obtained from AF2 model predictions on given Uniprot protein sequence data. Additionally, the Uniprot ID can be used to query binding site information from the Uniprot Database^54^. We obtain the union of binding sites for each Uniprot ID as the binding range (*start*, *end*). With the structure *S* from AF2, we can acquire the distance matrix *Dist* between all residue pairs, where *Dist*_*ij*_ is the distance between residue *i* and residue *j*. By thresholding, the contact map is obtained for each target:

1 $${C}_{ij}=\left\{\begin{array}{ll}1,\quad Dis{t}_{ij} < \,{\text{threshold}}\,\\ 0,\quad {\text{otherwise}}\,.\end{array}\right.$$

Here, we use a threshold of 8 Å, a common setting in protein structure analysis^55,56^. This threshold is applied to the distances between beta carbon atoms of residue pairs. By utilizing the binding range and the contact map, we can obtain the edge representation for the target binding site graph *E*_*t*_ = *C*[*start*: *end*, *start*: *end*]. The nodes *V*_*t*_ are the residues within the binding range.

### Sequence modality feature extraction module

After obtaining the token sets *Token*_*t*_ and *Token*_*d*_ for the target and drug through tokenization, independent embedding layers, and fully connected layers (FC) are utilized to acquire embedded representations of the drug and target, respectively:

2 $$\begin{array}{ll}Em{b}_{t}\,=\,FC(Embedding(Toke{n}_{t})),\\ Em{b}_{d}\,=\,FC(Embedding(Toke{n}_{d})).\end{array}$$

Then, we introduced the Group Enhance Module (GEM)^57^ to conduct inter-group feature enhancement on the embeddings:

3 $$\begin{array}{ll}E{F}_{t}\,=\,GE{M}_{t}(Em{b}_{t}),\\ E{F}_{d}\,=\,GE{M}_{d}(Em{b}_{d}).\end{array}$$

The GEM splits the input features ${\bf{X}}\in {{\mathbb{R}}}^{C\times L}$ into *G* groups and applies channel-wise enhancement to each group, where *C* is the number of channels and *L* is the length of the sequence. Specifically, for each group, the features are multiplied with averaged features over the channel dimension and then aggregated via summation. This results in a 1D modulation weight for each sample and group ${t}_{i,g}\in {{\mathbb{R}}}^{L}$. We further normalize *t*_*i*,*g*_ by subtracting the mean and dividing by the standard deviation plus a small constant *ϵ*. The normalized tensors are projected with learned parameters to obtain nonlinear modulation weights *σ*(*w*_*g*_ ⋅ *t*_*i*,*g*_ + *b*_*g*_), where *σ* refers to the sigmoid function.

Then, we applied BiLSTM modules on the enhanced features to enable bidirectional sequential interaction and extraction:

4 $$\begin{array}{ll}{F}_{t}\,=\,BiLSTM(E{F}_{t}),\\ {F}_{d}\,=\,BiLSTM(E{F}_{d}).\end{array}$$

To effectively model the interactions between drug and target representations, we proposed a multi-head link attention mechanism. Given the target features ${F}_{t}\in {{\mathbb{R}}}^{{L}_{t}\times D}$ and drug features ${F}_{d}\in {{\mathbb{R}}}^{{L}_{d}\times D}$, where *L*_*t*_ and *L*_*d*_ denote the lengths of the target and drug sequences, respectively, and *D* is the feature dimension, we first generate masking matrices ${M}_{t}\in {{\mathbb{R}}}^{H\times {L}_{t}}$ and ${M}_{d}\in {{\mathbb{R}}}^{H\times {L}_{d}}$ to avoid illegitimate attention, since the sequences can be of variable lengths.

Then, multi-head link-attention is applied to capture internal dependencies:

5 $$\begin{array}{lll}{\tilde{F}}_{t},{A}_{t}&=&{\text{LinkAttn}}\,({F}_{t},{M}_{t}),\\ {\tilde{F}}_{d},{A}_{d}&=&{\text{LinkAttn}}\,({F}_{d},{M}_{d}),\end{array}$$

where *A*_*t*_ and *A*_*d*_ are the attention matrices, and ${\tilde{F}}_{t}$ and ${\tilde{F}}_{d}$ are the enhanced feature representations.

To model inter-representation attention, the drug and target features are concatenated and fed into another multi-head self-attention module with concatenated masks:

6 $${\tilde{F}}_{{\rm{cat}}},{A}_{{\rm{cat}}}=\,{\text{LinkAttn}}({\rm{Concat}}({F}_{t},{F}_{d}),{\rm{Concat}}\,({M}_{t},{M}_{d})).$$

This multi-head link attention mechanism allows complex inter- and intra-representation interactions to be effectively modeled for drug-target affinity prediction.

The link attention module is implemented as:

7 $$\begin{array}{lll}\,{\text{Query}}\,&=&\,{\text{Linear}}\,(X),\\ E&=&\left\{\begin{array}{ll}\,{\text{Query}}\,,\quad &\,{\text{if}}\,\,{M}_{ij}=1\\ -\infty ,\quad &\,{\text{otherwise}}\,\end{array}\right.,\\ A&=&\,{\text{Softmax}}\,(E),\\ \,{\text{Output}}\,&=&\,{\text{MatMul}}\,(A,V),\end{array}$$

where $X\in {{\mathbb{R}}}^{L\times D}$ is the input feature, $M\in {{\mathbb{R}}}^{H\times L}$ is the masking matrix, and *V* = *X*. The linear projection layer transforms the input into the query vector $\,\text{Query}\,\in {{\mathbb{R}}}^{H\times L}$. The mask shields illegal positions with − *∞*, so the attention distribution $A\in {{\mathbb{R}}}^{H\times L}$ focuses only on valid positions. Finally, the attention distribution is multiplied by the original features to obtain the enhanced output.

Finally, the outputs of the three attention modules are concatenated to produce the final sequence representation *R*_*seq*_ containing both internal and cross-representation dependencies:

8 $${R}_{seq}=\,{\text{Concat}}\,({\tilde{F}}_{t},{\tilde{F}}_{d},\tilde{F}\,\text{cat}).$$

Following the multi-head link-attention layers, we apply a point-wise feed-forward network (FFN) to further enrich the feature representations:

9 $$Ou{t}_{seq}=\,{\text{FFN}}(x)={\text{ReLU}}\,(Wx+b)+x,$$

where *W* and *b* are learnable parameters of the linear transformation, and ReLU refers to the rectified linear unit activation function.

This FFN provides additional enhancement capability to the feature representations. Stacking multiple link-attention and FFN layers forms a powerful encoder for learning advanced representations.

### Graph modality feature extraction module

Through data retrieval and construction, we obtain the target graph *G*_*t*_ = (*V*_*t*_, *E*_*t*_) and drug molecular graph *G*_*d*_ = (*V*_*d*_, *E*_*d*_). To model the interactions between drugs and targets on graph neural networks, we introduce virtual nodes as connections between the two graphs. Specifically, a fused graph *G*_*f*_ = (*V*_*f*_, *E*_*f*_) will be constructed. The nodes are the union of nodes from both graphs and the virtual nodes:

10 $${V}_{f}={V}_{t}\cup {V}_{d}\cup {V}_{vir}.$$

The edge relations retain edges within each graph, while virtual nodes additionally connect all nodes from both graphs. This builds bridges for information flow between the two graphs:

11 $${E}_{f}=\left(\begin{array}{ccc}{E}_{t}&{\bf{1}}&{\bf{0}}\\ {\bf{1}}&1&{\bf{1}}\\ {\bf{0}}&{\bf{1}}&{E}_{d}\end{array}\right),$$

where **1** represents vectors of ones, 1 denotes the scalar value one, and **0** represents matrices of zeros.

Moreover, since targets and drugs are essentially different-level objects, the node features of the two graphs are not consistent. Therefore, simply connecting them via virtual nodes is unreasonable. Hence, we utilize the *MFE*_*seq*_ described from the previous section to endow both graphs with high-dimensional, homologous node features.

12 $$N{F}_{t},N{F}_{d}=MF{E}_{seq}({S}_{t},{S}_{d}),$$

where *NF*_*t*_ and *NF*_*d*_ are the node features of the target and drug graphs, respectively.

To directly correspond to the virtual node features, the averaged feature of the special “[EOS]” tokens for targets and drugs are assigned to the virtual nodes. This avoids the hierarchical clash between the two graphs.

Meanwhile, to make the extracted sequence-based features more characteristic of the targets and drugs, we proposed a warm-up strategy where the model is first trained on only the sequence part, allowing it to learn interaction patterns between drug-target pairs before assigning fusion graph node features for joint training. This ensures feature validity.

In addition, to facilitate model identification of node sources, nodes are endowed with an extra source type feature indicating whether the node is from the target graph, drug molecular graph, or a virtual node.

13 $${NF}^{{\prime} }=(NF,\,{\text{Source}}\,{\text{type}}).$$

The fused graph *G*_*f*_ is passed through a multi-layer graph neural network (MGNN) to learn hierarchical representations:

14 $$Ou{t}_{str}=\,{\text{MGNN}}({G}_{f})={\text{MGNN}}\,({V}_{f},{E}_{f}).$$

The MGNN contains *L* stacked Graph Isomorphism Network convolution (GINConv) layers, with each layer composed of a Graph Isomorphism Network (GIN)^58^ followed by batch normalization (BN):

15 $${\text{MGNN}}_{l}=\text{BN}({\text{GINConv}}({\text{Input}}^{(l)})),\quad l=1,\ldots ,L,$$

where Input^(*l*)^ denotes the input to the *l*-th layer, and MGNN_*l*_ refers to the output of the *l*-th layer.

Specifically, the GINConv layer operator is defined as:

16 $$\,{\text{GINConv}}\,({\bf{x}})=\,{\text{MLP}}\,\left({\bf{x}}+\sum _{j\in {\mathcal{N}}(i)},\,{\text{MLP}}\,({{\bf{x}}}_{j})\right),$$

where ${\mathcal{N}}(i)$ denotes the neighbors of node *i* and MLP refers to a multi-layer perceptron with ReLU activation that transforms node features.

After propagating through the MGNN layers, the node features are aggregated via summation to obtain the final graph-level representation *Out*_*str*_.

### Modality fusion and prediction

The graph representation *Out*_*str*_ is concatenated with the sequence-based representation *Out*_*seq*_:

17 $$Ou{t}_{con}=\,{\text{Concat}}\,(Ou{t}_{str},Ou{t}_{seq}).$$

This integrates both sequential and graph-structured features. The integrated representation *Out*_*con*_ is fed into a fusion FFN to predict drug-target binding affinity:

18 $${\hat{y}}_{d,t}=\,{\text{FFN}}\,(Ou{t}_{con}).$$

### Model implementation and experiment setting

We implemented the model using Python libraries including Pytorch^59^, PyG^60^, and RDKit^51^. We employed mean square error loss (MSELoss) as the loss function and used the Adam optimizer for parameter optimization. Details of the model training process and hyperparameter settings can be found in Supplementary Note 3.

For fair comparison of the model, we adopted a 5-fold cross-validation strategy which splits the dataset into five equal portions. In each fold, one portion is held out as the test set while the remaining four portions are further divided into training and validation sets with a 7:1 ratio. Hence, the data used for model training is split into training, validation, and test sets with a ratio of 7:1:2. By selecting different portions as the test set, the experiment is repeated five times, and the average and standard deviation of results are reported. To ensure a fair comparison, our model and other baseline methods use the same data-splitting scheme for performance evaluation.

### Datasets and metrics

To evaluate method performance, we conducted experiments on two widely used DTA datasets in SOTA DTA prediction methods: Davis^61^ and KIBA^62^. Both datasets provide drug SMILES sequences, target amino acid sequences, and experimentally measured binding affinities between drug-target pairs. We employed three independent metrics: mean square error (MSE), concordance index (CI)^63^, and mean reversion coefficient (${r}_{m}^{2}$) to enable robust performance evaluation. Details of the datasets and metrics used in this study are provided in Supplementary Note 4 and 5.

## Supplementary information

Supplymentary Material

## Data Availability

The data using in this study are publicly available and can be accessed at https://github.com/hehh77/DMFF-DTA.

## Code availability

The source code and data of this study are available at https://github.com/hehh77/DMFF-DTA. The web server for the DMFF-DTA tool is hosted on Hugging Face Spaces and can be accessed at https://huggingface.co/spaces/hehh197/DMFF-DTA.
