# Supplementary material for: Dual modality feature fused neural network integrating binding site information for drug target affinity prediction
Source: NPJ Digit Med. 2025 Jan 28;8:67. doi: 10.1038/s41746-025-01464-x (PMC11775287; doi:10.1038/s41746-025-01464-x)
Supplement: Supplementary file 1 — Supplymentary Material [file 41746_2025_1464_MOESM1_ESM.pdf]

Supplementary Materials for ‘**Dual Modality Feature Fused Neural Network Integrating Binding Site Information for Drug Target Affinity Prediction**’

Haohuai He[†1], Guanxing Chen[†1], Zhenchao Tang[1], and Calvin Yu-Chian Chen [*2,3,4,5]

[1]Artificial Intelligence Medical Research Center, School of Intelligent Systems Engineering, Shenzhen Campus of Sun Yat-sen University, Shenzhen, 518107, China

[2]AI for Science (AI4S)-Preferred Program, School of Electronic and Computer Engineering, Peking University Shenzhen Graduate School, Shenzhen, 518055, China

[3]State Key Laboratory of Chemical Oncogenomics, Key Laboratory of Chemical Genomics, School of Chemical Biology and Biotechnology, Peking University Shenzhen Graduate School, Shenzhen, 518055, China

[4]Department of Medical Research, China Medical University Hospital, Taichung, 40447, Taiwan

[5]Department of Bioinformatics and Medical Engineering, Asia University, Taichung, 41354, Taiwan

This PDF file includes:

- Supplementary Figures 1-2
- Supplementary Tables 1-11
- Supplementary Note 1-8

[*]Corresponding author. Email Address: cy@pku.edu.cn

[†]These authors contributed equally to this work.

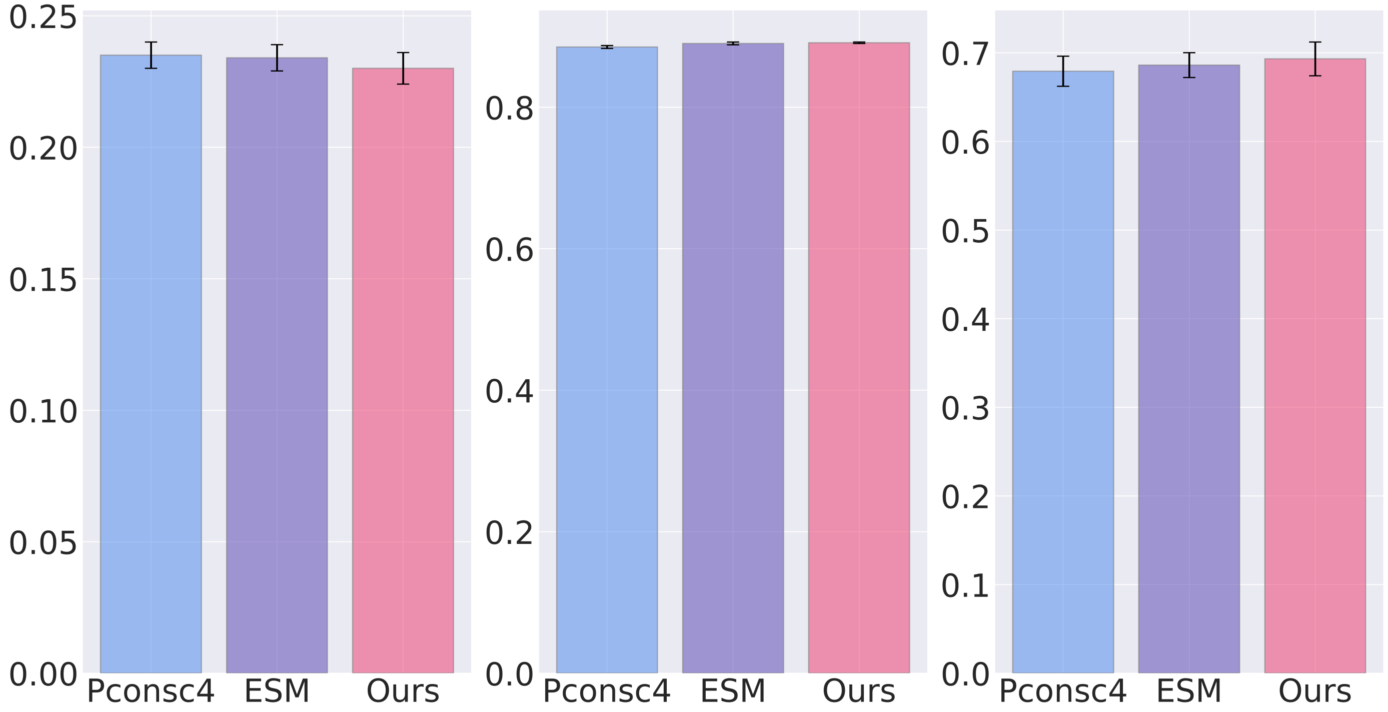

Supplementary Figure 1. Bar charts illustrating the performance of different contact map construction techniques for MSGNN-DTA on the Davis dataset. The metrics reported are MSE, CI, and r_m^2. Our AF2-based contact map generation method outperforms Pconsc4 and ESM, demonstrating its effectiveness.

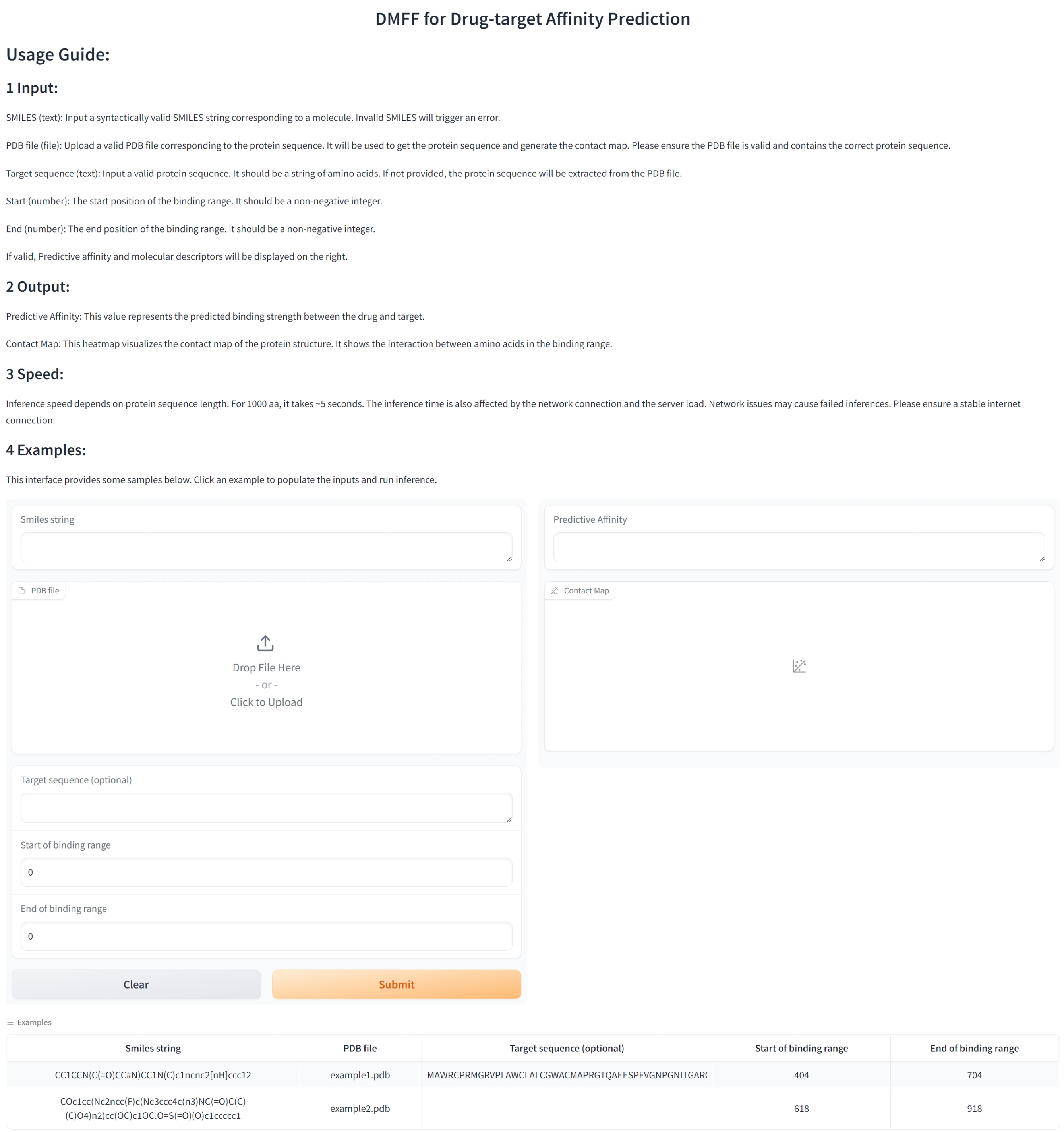

Supplementary Figure 2. Screenshot of the web server interface for DMFF-DTA. The web server allows users to input drug SMILES, protein PDB files, and target sequences to obtain drug-target affinity predictions.

Supplementary Table 1. Performance comparison (average $\pm$ std) of DMFF-DTA and other SOTA models on the Davis dataset. Bold indicates the best performance, and underline indicates the second best for each metric. Metrics reported are MSE, CI, and r_m^2. ↑/↓ indicates that the larger/smaller the metrics, the better the model performance.

Method	MSE ↓	CI↑	r_m^2↑
DeepDTA	0.284(0.008)	0.869(0.002)	0.624(0.015)
GraphDTA	0.242(0.010)	0.880(0.001)	0.683(0.010)
FusionDTA	<u>0.226(0.005)</u>	<u>0.891(0.001)</u>	0.686(0.020)
MgraphDTA	0.228(0.004)	0.883(0.002)	0.679(0.022)
MSGNN-DTA	0.234(0.005)	0.890(0.002)	0.686(0.014)
AttentionSiteDTI	0.238(0.007)	0.886(0.003)	0.683(0.021)
AttentionMGT	0.229(0.006)	0.888(0.002)	<u>0.690(0.017)</u>
DMFF(Ours)	**0.218(0.004)**	**0.894(0.002)**	**0.702(0.029)**

Supplementary Table 2. Performance comparison (average $\pm$ std) of DMFF-DTA and other SOTA models on the KIBA dataset. Bold indicates the best performance, and underline indicates the second best for each metric. Metrics reported are MSE, CI, and r_m^2. ↑/↓ indicates that the larger/smaller the metrics, the better the model performance.

Method	MSE ↓	CI↑	r_m^2↑
DeepDTA	0.201(0.003)	0.850(0.002)	0.671(0.017)
GraphDTA	0.192(0.001)	0.848(0.002)	0.718(0.004)
FusionDTA	0.155(0.002)	0.882(0.001)	0.750(0.015)
MgraphDTA	0.152(0.003)	0.884(0.002)	<u>0.766(0.016)</u>
MSGNN-DTA	<u>0.149(0.003)</u>	<u>0.885(0.002)</u>	0.763(0.009)
AttentionSiteDTI	0.157(0.004)	0.881(0.002)	0.759(0.016)
AttentionMGT	0.150(0.003)	0.879(0.001)	0.762(0.011)
DMFF(Ours)	**0.144(0.002)**	**0.889(0.002)**	**0.773(0.016)**

Supplementary Table 3. Performance and cost comparison of DMFF-DTA with different binding range settings on the Davis dataset. Metrics reported are runtime, MSE, and GPU consumption. The runtime is in seconds, and GPU consumption is in MB. The runtime indicates the time taken to train the model for one epoch. The GPU consumption denotes memory usage during training.

Range Length	Runtime	MSE	GPU Consumption
100	33.50	0.236	12305
200	35.21	0.233	15221
300	36.81	0.218	17681
400	38.33	0.222	21765
500	39.14	0.225	23691
600	38.74	0.222	25755
700	40.58	0.221	26557
800	43.07	0.224	29599
900	42.36	0.227	30131
1000	43.13	0.218	30573

Supplementary Table 4. Performance and cost comparison of DMFF-DTA and other SOTA models on the Davis dataset. Metrics reported are runtime, MSE, and GPU consumption. The runtime is in seconds, and GPU consumption is in MB. The runtime indicates the time taken to train the model for one epoch. The GPU consumption denotes memory usage during training. And the MSE is the mean squared error on the test set.

Methods	Runtime	MSE	GPU Consumption
FusionDTA	47.54	0.226	7246
MgraphDTA	22.76	0.228	3520
MSGNN-DTA	32.78	0.234	22340
AttentionMGT	321.59	0.229	21726
DMFF(Ours)	36.80	0.218	17681

Supplementary Table 5. The performance comparison (average $\pm$ std) of different contact map construction methods on the Davis dataset. Bold indicates the best performance. Metrics reported are MSE, CI, and r_m^2.

Methods	Techniques	MSE	CI	r_m^2
DMFF-DTA	Pconsc4	0.225 (0.006)	0.883 (0.002)	0.691 (0.015)
	ESM	0.222 (0.005)	0.891 (0.001)	0.698 (0.021)
	Ours	**0.218 (0.004)**	**0.894 (0.002)**	**0.702 (0.029)**
MSGNN-DTA	Pconsc4	0.235 (0.005)	0.885 (0.002)	0.679 (0.017)
	ESM	0.234 (0.005)	0.890 (0.002)	0.686 (0.014)
	Ours	**0.230 (0.006)**	**0.891 (0.001)**	**0.693 (0.019)**

Supplementary Table 6. Performance comparison (average $\pm$ std) between our model and SOTA methods on the pancreatic cancer dataset. DMFF achieves superior performance over other methods in all metrics, demonstrating its strong predictive capabilities.

Methods	MSE	CI	r_m^2
Fusion-DTA	0.219	0.872	0.836
Mgraph-DTA	0.217	0.875	0.833
MSGNN-DTA	0.220	0.877	0.838
AttentionMGT	0.219	0.874	0.836
DMFF(Ours)	**0.212**	**0.881**	**0.842**

Supplementary Table 7. Performance comparison of our model and Graph Transformer-based AI methods on DTA prediction. Comparison of MSE, CI, and r_m^2 metrics on Davis and KIBA datasets. Lower MSE is better, while higher values are better for the other two metrics. Bolded numbers indicate optimal performance for each metric. "NA" indicates the method was not successfully validated on the dataset.

Methods	Davis			KIBA		
	MSE	CI	r_m^2	MSE	CI	r_m^2
Graphormer	0.890	0.606	0.068	NA	NA	NA
Grover	0.256	0.876	0.641	0.149	0.883	0.758
DMFF(Ours)	**0.218**	**0.894**	**0.702**	**0.144**	**0.889**	**0.773**

Supplementary Table 8. Performance comparison of DMFF and other binding site-based methods on the Unknown Binding Pocket Scenarios. Metrics reported are MSE, CI, and r_m^2. Lower MSE is better, while higher values are better for the other two metrics. Bolded numbers indicate optimal performance for each metric.

Evaluate set	Methods	MSE	CI	r_m^2
Known Binding Pocket	AttentionSiteDTI	0.376(0.035)	0.819(0.017)	0.422(0.048)
	AttentionMGT	0.362(0.029)	0.820(0.013)	0.430(0.051)
	DMFF(Ours)	**0.335(0.027)**	**0.837(0.011)**	**0.496(0.035)**
Unknow Binding Pocket	AttentionSiteDTI	0.461(0.017)	0.774(0.005)	0.372(0.026)
	AttentionMGT	0.457(0.028)	0.786(0.007)	0.395(0.015)
	DMFF(Ours)	**0.438(0.033)**	**0.797(0.012)**	**0.401(0.032)**

Supplementary Table 9. Hyper-parameters Settin for DMFF-DTA. It includes training settings and model configurations.

	Hyper-parameter	Value
Training Setting	lr_{max}	1e-3
	lr_{min}	5e-4
	Number of training epochs	200
	Number of warm up epochs	100
	Mini-batch size	128
	T_{max}	20
	Activation function	ReLU
Model Configuration	Embedding dimension	256
	Enhanced group for drug	20
	Enhanced group for target	200
	BiLSTM hidden dimension	128
	BiLSTM layer	2
	Attention head number	8
	Attention head dimension	256
	Dropout rate	0.2
	Binding range length	300
	MGNN layer	5
	MGNN hidden dimension	128

Supplementary Table 10. Performance comparison of different sequence feature extraction methods on the Davis dataset. Comparison of MSE, CI, and r_m^2 metrics. Lower MSE is better, while higher values are better for the other two metrics.

Methods	MSE	CI	r_m^2
LSTM	0.228 (0.007)	0.888 (0.006)	0.680 (0.024)
BiGRU	0.223 (0.003)	0.894 (0.003)	0.694 (0.020)
Ours	**0.218 (0.004)**	**0.894 (0.002)**	**0.702 (0.029)**

Supplementary Table 11. Performance comparison between DMFF-Binary-Light and AutoDock Vina on the GraphDTI dataset. Results are reported as AUC scores (standard deviation in parentheses).

	GraphDTI dataset	
Methods	Random-split	Cluster-based
DMFF-Binary-Light	0.963(0.0012)	0.945(0.0017)
AutoDock Vina	0.534(0.0044)	0.551(0.0372)

Supplementary Note

1 Results for Comparison of DMFF-DTA and Graph Transformer-based Methods

Graph Transformer is a commonly used framework that fuses sequence and graph structure information to enable interactive information passing. By leveraging attention mechanisms, Graph Transformers can capture dependencies between various modalities while remaining computationally efficient compared to other graph learning techniques. Since proteins and drugs also have both sequence and graph structure modalities, many advanced Graph Transformer-based AI methods have been applied across various computational biology domains.

Therefore, we selected two state-of-the-art graph Transformer methods [1, 2] tailored for AI for drug design and compared them against our proposed model. All methods used 5-fold cross-validation, with further splits into training, validation, and test sets in a 7:1:2 ratio. Graph Transformer models utilized hyperparameters mentioned in the original papers. As Graphormer is designed for smaller molecular graphs, applying it to drug-target affinity prediction is resource-intensive and slow for large target graphs, hence it was only successfully validated on the Davis dataset.

Supplementary Table 7 shows the performance of different methods on the Davis and KIBA datasets. Our model outperforms the graph Transformer approaches on all metrics. Our method achieves lower MSE and higher CI and r_m^2 compared to Graphormer and Grover on the Davis dataset. Specifically, we improve MSE by 0.038 and 0.672 over Grover and Graphormer respectively. For the KIBA dataset, our method again demonstrates superior performance, reducing MSE by 0.015 compared to Grover. This may be because graph Transformers do not fully consider the characteristics of drugs and targets, and overlook modeling between them. Given these methods are not specifically designed for DTA prediction, it highlights the necessity of task-specific design and suggests graph Transformers may not be best suited for this particular task.

2 Comparative Analysis of DMFF and Binding Site-Based Methods on Unknown Binding Pocket Scenarios

To evaluate the performance of DMFF compared to binding site-based methods on unknown binding pocket scenarios, we designed an experiment that simulates real-world scenarios where binding pocket information may or may not be available. We divided the targets into two sets: one with known binding pockets and another with unknown binding pockets. The models were trained and validated on the dataset with known binding pockets and then further validated on the set with unknown binding pockets. We split the targets into two groups - known binding pockets and unknown binding pockets. All models (AttentionSiteDTI [3, 4], AttentionMGT [5], and our proposed DMFF) were trained on the dataset with known binding pockets. The models were then evaluated on both the known binding pocket set and the unknown binding pocket set. Supplementary Table 8 presents the performance metrics (MSE, CI, and r_m^2) for each model on both the known and unknown binding pocket sets.

As shown in Supplementary Table 8, DMFF consistently outperforms the binding site-based methods (AttentionSiteDTI and AttentionMGT) in both known and unknown binding pocket scenarios. The superior performance is evident across all metrics.

It is important to note that the overall performance of all models in this experiment is lower than previous results. This decrease in performance is attributed to the challenging nature of the experimental design. By dividing targets rather than randomly sampling, we created a scenario that closely resembles the "unseen target" problem, which is inherently more complex than random sample splitting.

In the known binding pocket set, DMFF achieves the lowest MSE (0.335), highest CI (0.837), and highest r_m^2 (0.496). This performance advantage is maintained in the unknown binding pocket set. Here, DMFF again outperforms the other methods across all metrics with an MSE of 0.438 (compared to 0.457 for AttentionMGT and 0.461 for AttentionSiteDTI), a CI of 0.797 (versus 0.786 for AttentionMGT and 0.774 for AttentionSiteDTI), and an r_m^2 of 0.401 (compared to 0.395 for AttentionMGT and 0.372 for AttentionSiteDTI). These results highlight DMFF's consistent superiority in both scenarios.

The consistent superior performance of DMFF, especially in the unknown binding pocket scenario, demonstrates its robustness and generalization capability. This result suggests that DMFF is better equipped to handle cases where binding site information is limited or unavailable. DMFF maintains its predictive power because it processes complete drug-target information through its architecture, where binding site information serves as supplementary guidance rather than prerequisite input. This design ensures that the model can effectively capture relevant interaction patterns even when specific binding pocket data is unavailable. Therefore, it is a more effective and widely applicable tool for predicting drug-target affinity across diverse contexts.

3 Training Configuration

For model training, we utilized mean squared error (MSE) as the loss function to minimize the discrepancy between predicted binding affinities $\hat{y}_{d,t}$ and ground-truth values $y_{d,t}$. A cosine annealing strategy was adopted to adjust the learning rate lr during training:

$$lr = lr_{min} + \frac{1}{2}(lr_{max} - lr_{min})(1 + \cos(\frac{T_{cur}}{T_{max}}\pi)) \tag{1}$$

where lr_{min} and lr_{max} are the lower and upper bounds for lr, and T_{max} is the total period that lr fluctuates cosmically. T_{cur} denotes the current epoch number. The Adam optimizer was used to update model parameters:

$$\theta_{t+1} = \theta_t - \frac{\eta}{\sqrt{\hat{v}t} + \epsilon}\hat{m}t \tag{2}$$

where θ_t is the parameter at epoch t, η is the learning rate, $\hat{m}t$ and $\hat{v}t$ are estimations of the first and second moment of the gradient, and ϵ is a small constant for numerical stability. The hyperparameter settings are summarized in Supplementary Table 9.

All experiments were performed on Linux workstations equipped with NVIDIA GeForce RTX A4000 and A6000 GPUs as well as Intel Xeon Silver 4210R 2.40GHz CPUs. The code was implemented in PyTorch and PyTorch Geometric.

4 Datasets

Our study utilizes two widely used datasets in drug-target affinity (DTA) prediction: Davis and KIBA. These datasets are chosen for their comprehensive coverage of kinase inhibitors and their targets and their ability to capture different aspects of drug-target interactions.

4.1 Davis Dataset

The Davis dataset, introduced by Davis et al. [6], provides a comprehensive analysis of kinase inhibitor selectivity. It contains interaction data for 72 kinase inhibitors tested against 442 kinases, covering over 80% of the human catalytic protein kinome. The dataset includes 30,056 drug-protein (DP) pairs, each comprising the SMILES representation of the drug, the amino acid sequence of the protein, and the IC50 activity value (converted to negative logarithm pIC50 for our analysis). This dataset is particularly valuable for studying the selectivity profiles of kinase inhibitors, including both type I and type II inhibitors. It allows for the identification of 'group-selective' inhibitors that are broadly active against a single subfamily of kinases but selective outside that subfamily. The comprehensive nature of the Davis dataset makes it an essential resource for developing and evaluating DTA prediction models, especially those focusing on kinase inhibitors.

4.2 KIBA Dataset

The KIBA (Kinase Inhibitor BioActivity) dataset, developed by Tang et al. [7], addresses the challenge of integrating heterogeneous bioactivity data. It contains 118,083 DP pairs, consisting of 2,068 drugs and 229 proteins. Each data point includes the SMILES representation of the drug, the amino acid sequence of the protein, and a KIBA score as the binding activity value. The KIBA score is derived from a model-based ensemble approach that integrates different types of bioactivity data (IC50, Ki, and Kd). This integration allows for a more comprehensive representation of drug-target interactions, capturing complementary information from various experimental assays. The KIBA dataset's unique approach to combining multiple bioactivity types makes it particularly useful for developing models that can handle diverse forms of interaction data, potentially leading to more robust and versatile DTA prediction methods.

4.3 Significance and Challenges in DTA Prediction

The Davis and KIBA datasets are widely used in DTA prediction due to their comprehensive coverage, high data quality, and diverse information. The Davis dataset's focus on kinase inhibitors and the KIBA dataset's integrated bioactivity scores offer complementary perspectives on drug-target interactions. Despite their extensive use, these datasets still present challenges for many models, indicating unresolved issues in DTA prediction. Common difficulties include handling the data imbalance caused by the significant size difference between drugs and proteins, effectively incorporating protein structural information, and integrating diverse features from both sequence and structural data. Our DMFF-DTA model addresses these challenges through a binding site-focused approach using AF2 and data mining to construct more balanced and informative target graphs. The model's dual-modal architecture effectively integrates sequence and graph modality information, while its innovative feature fusion and balancing techniques improve the ability to capture complex drug-target interactions. Using these datasets, we demonstrate DMFF-DTA's ability to overcome common challenges in DTA prediction and showcase its potential for improving drug discovery and repositioning efforts. The performance of DMFF-DTA on these well-established datasets underscores its effectiveness in addressing critical limitations of existing methods and its potential to advance the field of drug-target affinity prediction.

5 Metrics

5.1 Mean Squared Error (MSE)

To evaluate model prediction performance, we employed mean squared error (MSE) as an evaluation metric. MSE measures the average squared difference between the predicted binding affinities $\hat{y}_{d,t}$ and the ground-truth experimental measurements $y_{d,t}$:

$$MSE = \frac{1}{N}\sum_{i=1}^{N}(\hat{y}_{d,t} - y_{d,t})^2, \tag{3}$$

where N is the number of drug-target pairs in the dataset. MSE amplifies larger errors due to the squaring term, making it more sensitive to outliers compared to MAE. A lower MSE indicates better model performance. We utilize MSE here as it directly measures the gap between predictions and ground truth based on the scale of the target values.

5.2 Mean Reversion Coefficient (r_m^2)

To assess the generalization ability of our model, we utilized the mean reversion coefficient (r_m^2) as an evaluation metric. r_m^2 measures how well the predicted binding affinities $\hat{y}_{d,t}$ correlate to the experimentally measured values $y_{d,t}$ on external test data:

$$r_m^2 = (1 - \sqrt{(r^2 - r_0^2)}) * r^2, \tag{4}$$

where r is the squared correlation coefficient between $\hat{y}_{d,t}$ and $y_{d,t}$ with intercept, and r_0 is the correlation without intercept. r_m^2 discounts the correlation contributed by the intercept to focus on the remaining correlation indicative of the model's generalization ability. Higher r_m^2 suggests better extrapolation on new data, with 1 being perfect correlation.

5.3 Concordance Index (CI)

The concordance index (CI) measures the correctness of the relative order between predicted binding affinities $\hat{y}_{d,t}$ and experimentally measured values $y_{d,t}$. The CI is calculated as:

$$CI = \frac{1}{Z}\sum_{y_{d,t_i} > y_{d,t_j}} h(\hat{y}_{d,t_i} - \hat{y}_{d,t_j}), \tag{5}$$

where $\hat{y}_{d,t_i}$ and $\hat{y}_{d,t_j}$ are predicted binding affinities for sample i and j, y_{d,t_i} and y_{d,t_j} are the corresponding experimental measurements, Z is a normalization constant, and $h(x)$ is the step function defined as:

$$h(x) = \begin{cases} 1 & \text{if } x > 0 \\ 0.5 & \text{if } x = 0 \\ 0 & \text{if } x < 0 \end{cases}. \tag{6}$$

The CI evaluates if the model correctly orders sample pairs with higher and lower binding affinities. A CI of 1 indicates perfect concordance, while 0.5 is equivalent to random ranking.

6 Web Server for DMFF-based Drug-Target Affinity Prediction

To facilitate the use of our DMFF-DTA model for drug-target affinity prediction, we have developed a user-friendly web server. This web server is built on the Hugging Face Spaces platform, a cloud-based environment that enables the deployment of machine learning models as web services. It also based on the Gradio library, which provides a simple interface for creating web-based applications for machine learning models. This server allows researchers to easily input molecular data and obtain affinity predictions without the need for local computational resources or complex software installations.

6.1 Interface and Usage

The web server provides a simple and intuitive graphical user interface (Supplementary Figure 2). Users can input the following data:

- SMILES string: A text representation of the drug molecule's structure.
- PDB file: The protein structure file in PDB format.
- Target sequence (optional): The amino acid sequence of the target protein.
- Binding range: The start and end positions of the binding site on the protein sequence.

Upon submission, the server processes the input and returns two main outputs:

- Predictive Affinity: A numerical value representing the predicted binding strength between the drug and target.
- Contact Map: A heatmap visualization of the protein's contact map, focusing on the specified binding range.

The web server is designed for efficiency, with inference times typically around 5 seconds for a protein sequence of 1000 amino acids. However, actual performance can vary based on server load and network conditions.

6.2 Accessibility

The web server is freely accessible at https://huggingface.co/spaces/hehh197/DMFF-DTA. It is compatible with standard web browsers and does not require any additional software installation. Just make sure you can connect to the huggingface website. This makes our DMFF model easily accessible to researchers worldwide, promoting its use in drug discovery and related fields.

By providing this web server, we aim to make our DMFF model more accessible and user-friendly for the scientific community, allowing for quick and easy drug-target affinity predictions without the need for extensive computational resources or programming knowledge.

7 Comparison of Sequence Feature Extraction Methods

To address the effectiveness of our sequence feature extraction method, we conducted additional experiments comparing our approach with two commonly used sequence processing techniques: unidirectional Long Short-Term Memory (LSTM) and Bidirectional Gated Recurrent Unit (BiGRU). These experiments aim to demonstrate the efficacy of our proposed method in extracting meaningful features from drug and target sequences.

We replaced our sequence feature extraction module with LSTM and BiGRU implementations while keeping all other components of the DMFF model unchanged. We then evaluated the performance of these modified models on the Davis dataset.

As shown in Supplementary Table 10, our proposed sequence feature extraction method outperforms LSTM and BiGRU across all three metrics. Specifically, our method achieves the lowest Mean Squared Error (MSE) of 0.218, the highest Concordance Index (CI) of 0.894, and the best r_m^2 value of 0.702. The LSTM-based extraction method shows the weakest performance among all three methods, with the highest MSE (0.228) and lowest CI (0.888) and r_m^2 (0.680) values. The BiGRU-based method performs better than LSTM but still falls short of our proposed approach in all metrics.

These results demonstrate that our sequence feature extraction method is more effective in capturing relevant information from drug and target sequences compared to those approaches. The improved performance can be attributed to our method's ability to better capture long-range dependencies and relevant structural information from the input sequences.

8 Comparison with Molecular Docking Approach on GraphDTI Dataset

To enable comparison with molecular docking approaches, we followed the experimental protocol established in GraphDTI [8], where AutoDock Vina [9] was used as one of the baseline methods. Following their protocol, we developed DMFF-Binary-Light, a lightweight variant of DMFF modified for binary classification tasks like Drug-target Interaction (DTI). This model retains only the feature extraction components of the original DMFF architecture while replacing the regression output with a binary classifier.

We evaluated our approach on the GraphDTI benchmark dataset [8], consisting of 681,552 drug-target pairs. Following their validation scheme, we performed both random-split and cluster-based cross-validation. The cluster-based validation scheme was specifically designed to minimize the similarity between training and testing sets, providing a more rigorous assessment of model generalization. For AutoDock Vina evaluation, drug molecules were docked to binding pockets identified in target proteins with eFindSite, and proteins were ranked based on the computed binding energies. Model performance was evaluated using the area under the receiver operating characteristic curve (AUC).

Supplementary Table 11 presents the comparative results of DMFF-Binary-Light against AutoDock Vina. Our DMFF-Binary-Light significantly outperformed AutoDock Vina, achieving AUC scores of 0.963 and 0.945 for random-split and cluster-based setting, respectively. In contrast, the molecular docking approach using AutoDock Vina achieved AUC scores of 0.534 and 0.551. This substantial performance gap between deep learning-based methods and molecular docking approaches aligns with the observations reported in the previous studies. Although DMFF-Binary-Light shows strong performance, our results do not reach the near-perfect AUC scores reported by GraphDTI (which achieved 0.999 and 0.996 for random-split and cluster-based setting), which is reasonable as GraphDTI was specifically designed and optimized for the DTI prediction task. Despite this, DMFF-Binary-Light maintained robust performance even under the more challenging cluster-based validation setting, demonstrating its strong generalization capability.

Supplementary References

[1] Y. Rong, Y. Bian, T. Xu, W. Xie, Y. Wei, W. Huang, and J. Huang, "Self-supervised graph transformer on large-scale molecular data," *Advances in Neural Information Processing Systems*, vol. 33, pp. 12559–12571, 2020.

[2] C. Ying, T. Cai, S. Luo, S. Zheng, G. Ke, D. He, Y. Shen, and T.-Y. Liu, "Do transformers really perform badly for graph representation?," *Advances in Neural Information Processing Systems*, vol. 34, pp. 28877–28888, 2021.

[3] M. Yazdani-Jahromi, N. Yousefi, A. Tayebi, E. Kolanthai, C. J. Neal, S. Seal, and O. O. Garibay, "Attentionsitedti: an interpretable graph-based model for drug-target interaction prediction using nlp sentence-level relation classification," *Briefings in Bioinformatics*, vol. 23, no. 4, p. bbac272, 2022.

[4] N. Yousefi, M. Yazdani-Jahromi, A. Tayebi, E. Kolanthai, C. J. Neal, T. Banerjee, A. Gosai, G. Balasubramanian, S. Seal, and O. Ozmen Garibay, "Bindingsite-augmenteddta: enabling a next-generation pipeline for interpretable prediction models in drug repurposing," *Briefings in Bioinformatics*, vol. 24, no. 3, p. bbad136, 2023.

[5] H. Wu, J. Liu, T. Jiang, Q. Zou, S. Qi, Z. Cui, P. Tiwari, and Y. Ding, "Attentionmgt-dta: A multi-modal drug-target affinity prediction using graph transformer and attention mechanism," *Neural Networks*, vol. 169, pp. 623–636, 2024.

[6] M. I. Davis, J. P. Hunt, S. Herrgard, P. Ciceri, L. M. Wodicka, G. Pallares, M. Hocker, D. K. Treiber, and P. P. Zarrinkar, "Comprehensive analysis of kinase inhibitor selectivity," *Nature biotechnology*, vol. 29, no. 11, pp. 1046–1051, 2011.

[7] J. Tang, A. Szwajda, S. Shakyawar, T. Xu, P. Hintsanen, K. Wennerberg, and T. Aittokallio, "Making sense of large-scale kinase inhibitor bioactivity data sets: a comparative and integrative analysis," *Journal of Chemical Information and Modeling*, vol. 54, no. 3, pp. 735–743, 2014.

[8] G. Liu, M. Singha, L. Pu, P. Neupane, J. Feinstein, H.-C. Wu, J. Ramanujam, and M. Brylinski, "Graphdti: a robust deep learning predictor of drug-target interactions from multiple heterogeneous data," *Journal of cheminformatics*, vol. 13, pp. 1–17, 2021.

[9] O. Trott and A. J. Olson, "Autodock vina: improving the speed and accuracy of docking with a new scoring function, efficient optimization, and multithreading," *Journal of computational chemistry*, vol. 31, no. 2, pp. 455–461, 2010.
